# Establishment of six new *Rhabdoblatta* species (Blattodea, Blaberidae, Epilamprinae) from China

**DOI:** 10.3897/zookeys.851.31403

**Published:** 2019-06-03

**Authors:** Rong Yang, Zhenzhen Wang, Yanshuang Zhou, Zongqing Wang, Yanli Che

**Affiliations:** 1 Institute of Entomology, College of Plant Protection, Southwest University Beibei China; 2 Southwest University, Southwest University Beibei China; 3 Beibei, Chongqing 400715, China Southwest University Beibei China

**Keywords:** ABGD, female genitalia, GMYC, new species, species delimitation

## Abstract

This study examined 504 *Rhabdoblatta* specimens sampled from China, of which, 86 *Rhabdoblatta* specimens were used for COI sequencing. A phylogenetic analysis using the ML method and MOTUs estimations by ABGD and GMYC based on COI sequences was performed. Eighteen *Rhabdoblatta* species were identified when these data were combined with morphological data. Six new species were established among these samples, i.e., *Rh.similsinuata***sp. n.**, *Rh.densimaculata***sp. n.**, *Rh.gyroflexa***sp. n.**, *Rh.chaulformis***sp. n.**, *Rh.maculata***sp. n.**, and *Rh.ecarinata***sp. n.** For the first time, females including female genitalia of 14 known *Rhabdoblatta* species are described worldwide. Our study shows that combining molecular species delimitation methods with morphological data helps to delimit species and understand cockroach biodiversity.

## Introduction

Kirby established the genus *Rhabdoblatta* in 1903 and designated *Epilamprapraecipua* Walker, 1868 as the type species. After the work of many researchers (Shelford 1910; Hanitsch 1915; Bey-Bienko 1950; Princis 1967; Anisyutkin 2000, 2003, 2014), there are now more than 150 species in the largest genus *Rhabdoblatta* in Epilamprinae. Of these, 51 species are from China (Beccaloni 2014). Princis (1958) treated *Polyzosteriaterranea* Walker, 1868 as the synonym of *Epilamprapraecipua* Walker, 1868, but the type species of *P.terranea* was verified to be a nymph of Epilamprinae when examined by Anisyutkin (2014).

Anisyutkin (2003) divided 22 *Rhabdoblatta* species from Vietnam and Southern China into three groups based on the shape of the apical sclerite of L2D and the sclerite L3 hook: the *Rhabdoblattaklossi* group, the *Rhabdoblattaabdominalis* group and the *Rhabdoblattaelegans* group. Other members of *Rhabdoblatta* exhibit wide variation in the apical sclerite of L2D and sclerite L3 hook (Yang R, pers. obs.), indicating they can’t be arranged into any of the above groups. For example, *Rh.monticola*, *Rh.ecarinata* sp. n., *Rh.saussurei*, and *Rh.densimaculata* sp. n. should be placed in the *Rh.klossi* group based on the similar shape of the sclerite L3 hook, which is short, small and without any groove. However, the sclerite L2D of both *Rh.saussurei* and *Rh.densimaculata* sp. n. has the cap-shaped apical membrane and that of the other two species does not. These two could not therefore be assigned into any one of these three species groups. It was inferred from Legendre et al. (2017) and Wang (2018) using molecular data that the genus *Rhabdoblatta* was not a monophyletic group, and consists of members with distant relationships. The genus *Rhabdoblatta* still needs further revision; it is early to make a conclusion on the division of *Rhabdoblatta* species groups.

Since Hebert et al. (2003) came up with the conception of DNA barcodes; this methodology has gained wide acceptance as a supplementary method to identify species. This technique has been proven to be highly informative and to successfully resolve problems of polymorphism, sexual dimorphism and the identification of nymphs in cockroaches (Yue et al. 2014; Che et al. 2017; Bai et al. 2018; Evangelista et al. 2013). Two species delineation methods, Automatic Barcode Gap Discovery (ABGD) (Puillandre et al. 2012) and the General Mixed Yule-coalescent (GMYC) (Pons et al. 2006; Monaghan et al. 2009; Fujisawa and Barraclough 2013), applying the single-locus data to delimit species, have become the most popular approaches in DNA barcoding studies (Yue et al. 2014; Che et al. 2017; Bai et al. 2018; Evangelista et al. 2013).

To date, *Rhabdoblatta* species were described primarily on the basis of morphological characters and DNA barcoding has not been employed to investigate the diversity of *Rhabdoblatta*. In order to infer the diversity of *Rhabdoblatta* and resolve the issues of sexual dimorphism and matching nymphs, we generated new COI sequence data from a wide variety of representatives of this group combined, combined it with published data, and performed phylogenetic analyses, including ABGD and GMYC.

## Materials and methods

### Morphological study

Terminologies of male genitalia mainly follow Klass (1997) and Anisyutkin (2014). Genitalia abbreviations in the figures are as follows:

**R1**, **R2**, **R3**, **R4**, **R5**, **L2D** and **L3** sclerite of the male genitalia;

**IX** the ninth abdominal tergite;

**X** the tenth abdominal tergite;

**teVIII.** tergal process of the eighth abdominal tergite;

**teIX.** tergal process of the ninth abdominal tergite;

**V.I.** first valves of ovipositor;

**V.II.** second valves of ovipositor;

**V.III.** third valves of ovipositor;

**gg.** gonangulum of the female genitalia;

**pl.** sclerotized lobes of the second and third pairs of valves in the female genitalia;

**a.a.** anterior arch of second valvifer of the female genitalia;

**bsv.** basivalvula of the female genitalia;

**vs.** vestibular sclerite in the female genitalia;

**t.s.p.** transverse sclerotized plate in the female genitalia;

**bd.s.** brood sac of the female genitalia.

Measurements are based on specimens examined. The genital segments of the examined specimens were soaked in 10% NaOH, and then stored in glycerin for observation. All segments observed in glycerin jelly using a Motic K400 stereomicroscope. Photographs of the genitalia and body parts were taken using a Leica M205A stereomicroscope with Leica DFC Camera. Specimens were photographed using a Canon 50D with a Canon EF100mm f/2.8L Macro IS USM lens, and stacked with Helicon Focus software. All photos and images were edited with Adobe Photoshop CS5. The type materials are deposited in the Institute of Entomology, College of Plant Protection, Southwest University, Chongqing, China (SWU).

### PCR amplification and sequencing

The hind legs were used for molecular studies, and the other body parts were stored in 95% ethanol as voucher specimens. In total, 86 specimens were used for COI sequencing in this study and all sequences are deposited at the National Center for Biotechnology Information GenBank (Table 1).

**Table 1.** Samples used in species delimitation: sample collection localities, specimen voucher, and GenBank accession numbers.

Species	Location	Accession Number (specimen voucher)
* Rh. monticola *	Jiulonghu Lake, Guangdong	MK547352 (RhabMont01)
	Dayaoshan, Guangxi	MK547353 (RhabMont02), MK547354 (RhabMont03)
	Dinghushan, Guangdong	MK547355 (RhabMont04)
*Rh.ecarinata* sp. n.	Yinggeling, Hainan	MK547356 (Rhabcari01)
	Diaoluoshan, Hainan	MK547357 (Rhabcari02), MK547358 (Rhabcari03)
* Rh. atra *	Dayaoshan, Guangxi	MK547359 (RhabAtra01), MK547361 (RhabAtra03)
	Longtan Park, Guangxi	MK547360 (RhabAtra02)
* Rh. rattanakiriensis *	Wuzhishan, Hainan	MK547363 (RhabRatt01), MK547362 (RhabRatt02)
	Diaoluoshan, Hainan	MK547364 (RhabRatt03)
	Jianfengling, Hainan	MK547365 (RhabRatt04)
* Rh. elegans *	Jinzhongshan, Guangxi	MK547366 (RhabEleg01)
	Mengla, Yunnan	MK547367 (RhabEleg02)
	Baoshan, Yunnan	MK547368 (RhabEleg05)
* Rh. nigrovittata *	Gulin, Sichuan	MK547371 (RhabNigr01), MK547370 (RhabNigr02)
	Nanling, Guangdong	MK547372 (RhabNigr03)
	Simianshan, Chongqing	MK547373 (RhabNigr04), MK547375 (RhabNigr08)
	Dayaoshan, Guangxi	MK547374 (RhabNigr07)
	Shengtangshan , Guangxi	MK547376 (RhabNigr09)
	Tianmushan, Zhejiang	MK547377 (RhabNigr10)
	Mangshan, Hunan	MK547369 (RhabNigr11)
	Emeishan, Sichuan	MK547379 (RhabNigr12)
	Damingshan, Guangxi	MK547378 (RhabNigr13)
* Rh. simulans *	Medog, Xizang	MK547437 (RhabSimu02), MK547436 (RhabSimu05)
* Rh. marginata *	Jianfengling, Hainan	MK547381 (RhabMarg01), MK547380 (RhabMarg02)
	Wuzhishan, Hainan	MK547382 (RhabMarg03), MK547390 (RhabMarg11)
	Limushan, Hainan	MK547383 (RhabMarg04), MK547384 (RhabMarg05)
	Wuzhishan Scenic, Guangdong	MK547385 (RhabMarg06), MK547386 (RhabMarg07)
	Maogan, Hainan	MK547387 (RhabMarg08), MK547388 (RhabMarg09)
	Shengtangshan, Guangxi	MK547389 (RhabMarg10)
	Bawangling, Hainan	MK547391 (RhabMarg12)
* Rh. sinuata *	Butterfly Valley, Yunnan	MK547392 (RhabSinu01), MK547393 (RhabSinu02)
	Daheishan, Sichuan	MK547394 (RhabSinu03), MK547395 (RhabSinu04)
* Rh. mascifera *	Mengla, Yunnan	MK547407 (RhabMasc01)
	Menglun, Yunnan	MK547408 (RhabMasc02)
* Rh. incisa *	Ailaoshanshan, Yunnan	MK547399 (RhabInci01), MK547400 (RhabInci03)
	Daweishan, Yunnan	MK547401 (RhabInci04)
* Rh. krasnovi *	Maandi Village, Yunnan	MK547409 (RhabKras01), MK547411 (RhabKras02), MK547410 (RhabKras03), MK547412 (RhabKras04)
	Daweishan, Yunnan	MK547413 (RhabKras05)
* Rh. melancholica *	Diaoluoshan, Hainan	MK547425 (RhabMela01), MK547422 (RhabMela12), MK547421 (RhabMela13)
	Dayaoshan, Guangxi	MK547426 (RhabMela02)
	Dabieshan, Hubei	MK547427 (RhabMela03), MK547428 (RhabMela04)
	Huangshan, Anhui	MK547429 (RhabMela05)
	Kuankuoshui, Guizhou	MK547431 (RhabMela06), MK547430 (RhabMela07)
	Simianshan, Chongqing	MK547417 (RhabMela08), MK547418 (RhabMela09)
	Qingchengshan, Sichuan	MK547419 (RhabMela10), MK547420 (RhabMela11)
	Tianmushan, Zhejiang	MK547423 (RhabMela14), MK547424 (RhabMela15)
	Jinxiu, Guagnxi	MK547432 (RhabMela17)
* Rh. bicolor *	Jiangshan, Zhejiang	MK547414 (RhabBico01), MK547415 (RhabBico02)
	Huangshan, Anhui	MK547416 (RhabBico03)
* Rh. saussurei *	Huanjing, Guangxi	MK547434 (RhabSaus02)
	Mengla, Yunan	MK547433 (RhabSaus01)
*Rh.similsinuata* sp. n.	Ailaoshan, Yunnan	MK547397 (RhabSimi01), MK547396 (RhabSimi02), MK547398 (RhabSimi03)
*Rh.densimaculata* sp. n.	Jiguanshan, Sichuan	MK547402 (Rhabdens01)
	Ya’an, Sichuan	MK547403 (Rhabdens02), MK547404 (Rhabdens03)
	Ailaoshan, Yunnan	MK547405 (Rhabdens04)
	Medog, Xizang	MK547406 (RhabDens05)
*Rh.maculata* sp. n.	Leigongshan, Guizhou	MK547435 (RhabMacu)
*Rh.* sp. 3		MF804773 (RhabSp)
* Rh. atra *		KF640066 (RhabAtra)
* Rh. bielawskii *		KF640067 (RhabBiel)
*Rh.* sp. 1		KY497676 (RhabBl140)
*Rh.* sp. 2		KY497678 (RhabBl148)
*Rh.* sp. 4		KY497684 (RhabBl7)
* Mantis religiosa *		KR148854
		KM529415

The extraction procedure was according to the Hipure Tissue DNA Mini Kit. Total DNA was stored at −20 °C. Primers for the amplifications are COI-F_3_ (5’-CAACYAATCATAAAGANATTGGAAC-3’) and COI-R_3_ (5’-TAAACTTCTGGRTGACCAAARAATCA-3’). Each PCR was performed in Analytik Jena Easy Cycler with 25μL volumes using the aforementioned primers. The amplified samples were tested using agarose gel electrophoresis and sent for sequencing at BGI Technology Solutions Company Limited (BGI-Tech) (Beijing, China). All voucher specimens are deposited in the Institute of Entomology, College of Plant Protection, Southwest University, Chongqing, China.

### Sequences processing and phylogenetic analyses

A total of 94 COI sequences was analyzed (86 sequences representing *Rhabdoblatta* species by our own study and six sequences downloaded from GenBank, and two sequences representing the mantis outgroup downloaded from GenBank) (Table 1). Wang (2018) tried to use Blattellidae as outgroup, because Blattellidae is close to Blaberidae; the result is that Blattellidae inserts into the ingroup Blaberidae, and the topology is disorderly. Therefore, we chose the mantis as outgroup. All COI sequences were aligned using MEGA 7.0 and adjusted visually after translation into amino acid sequences. Intraspecific and interspecific genetic divergence values were quantified based on the Kimura 2-parameter (K2P) distance model (Kimura 1980), and variance was estimated by using the bootstrap method with 1000 bootstrap replications in MEGA 7.0 (Kumar et al. 2016). Maximum Likelihood (ML) analysis was implemented in RAxML 7.3.0 (Stamatakis et al. 2008) using GTRGAMMA model with 1000 bootstrap replicates.

We performed two molecular species delimitation methods, the Automatic Barcode Gap Discovery (ABGD: Puillandre et al. 2012) and the General Mixed Yule-coalescent (GMYC: Pons et al. 2006), in order to estimate the number of molecular operational taxonomic units (MOTUs) from the genus *Rhabdoblatta*. Automatic Barcode Gap Discovery (ABGD) was available at the web interface (http://wwwabi.snv.jussieu.fr/ public/abgd/) and was used as a simple, quick and efficient method with the default settings by Jukes-Cantor (JC69) and p distance model with a relative gap width (X = 1.0), it used the 92 COI sequences (excluding outgroups). The GMYC method requires a fully-resolved ultrametric tree for the analysis to define species. Time-resolved gene trees were inferred in BEAST 1.8.1 (Drummond and Rambaut 2007) using the best models from PartitionFinder V1.1.1 (Lanfear et al. 2012). The best-fitting models were as follows: COI_pos1, TrN+G; COI_pos2, TrN+G; COI_pos3, TrN+I. The following settings were used: rate variation was modeled among branches using a strict clock model with the mean clock rate fixed to 1 and the Birth-Death speciation was used as a tree prior. We then selected the GMYC method to the ultrametric gene tree using the SPLITS package (Ezard et al. 2009) in R (R Core Team 2013). The species delimited were compared to a one species null model using a likelihood ratio test. It used 70 COI sequences (the exact same sequence is left with only one) and excluding outgroups.

## Results

### Morphological delimitation of *Rhabdoblatta*

On the basis of morphological characters including male genitalia, we were able to identify 20 morphospecies of *Rhabdoblatta* among the 504 samples from China that we examined. Herein six new species, *Rh.similsinuata* sp. n., *Rh.densimaculata* sp. n., *Rh.gyroflexa* sp. n., *Rh.chaulformis* sp. n., *Rh.maculata* sp. n., and *Rh.ecarinata* sp. n. are established only according to morphological characters including male genitalia (Figs 2–7). Species descriptions are provided below.

### Phylogenetic analysis based on COI and MOTUs estimation

**Figure 1.**
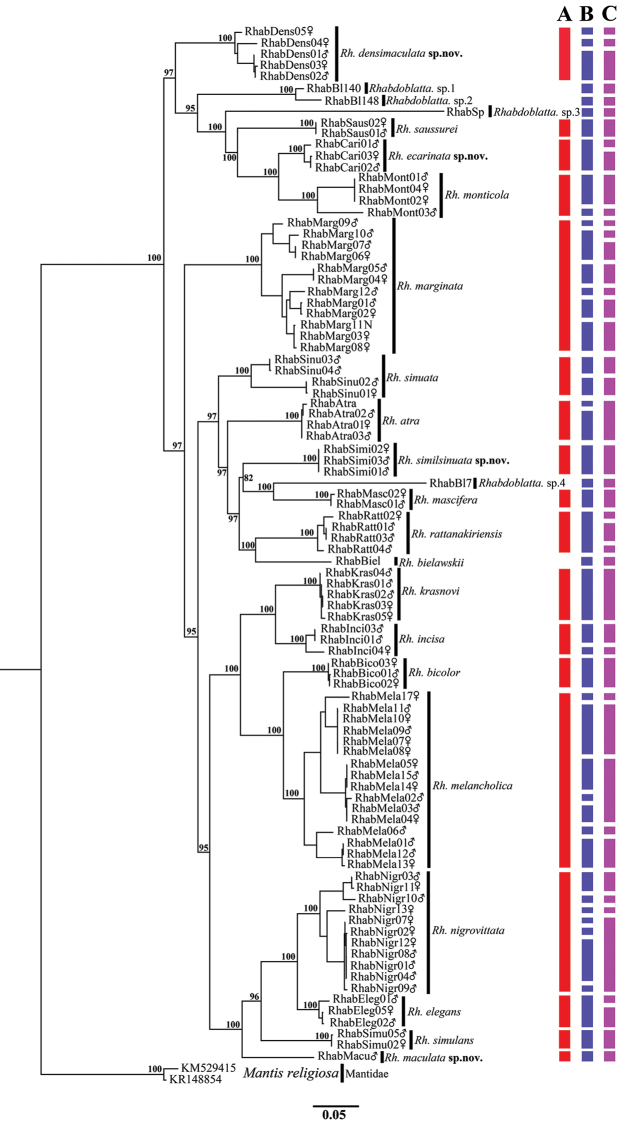
Maximum likelihood (ML) tree derived from COI gene analysis following GTR GAMMA model with 1000 bootstrap replicates. In red, referring to the morphospecies; in blue, referring to MOTUs in ABGD; in purple, referring to MOTUs in GMYC.

In this study, we acquired 86 *Rhabdoblatta* COI sequences representing 18 *Rhabdoblatta* morphospecies (other two morphospecies without molecular data), 83 of which, length excluding primers, were 658bp, the remaining were 619bp, 621bp and 634bp respectively. The COI region we sequenced had a relatively high AT content (66.3%) with an average nucleotide composition of A = 30.1%, T = 36.2%, C = 17.6%, and G = 16.1%. Sequence analysis revealed that 266 (40.30%) sites were variable, of which 243 (36.81%) sites were parsimoniously informative. ML analysis revealed that clades from the same morphospecies, including females and nymphs, constituted monophyletic groups (Figure 1).

We used two molecular species delimitation methods (ABGD, GMYC) in our study to delimit *Rhabdoblatta* samples. These two methods have yielded significantly different results using COI data; ABGD produced 46 MOTUs and GMYC 45 MOTUs respectively (Figure 1). ABGD analysis for MOTUs detection was estimated with JC69 and P = 0.001, and the likelihoods of the null and GMYC models from COI analysis were 264.71 and 281.99 respectively. The six sequences (downloaded from GenBank) provided six MOTUs by ABGD and five MOTUs by GMYC: RhabAtra and data of this study (RhabAtra01, RhabAtra02, RhabAtra03) resulted in one MOTU in GMYC, while two MOTUs in ABGD (RhabAtra were recovered as a single MOTU); the remaining five sequences were recovered as single MOTU under both methods. The same MOTUs were detected for remaining data (12 morphospecies) in both ABGD and GMYC analysis: *Rh.similsinuata* sp. n., *Rh.sinuata*, *Rh.atra*, *Rh.mascifera*, *Rh.simulans*, *Rh.bicolor*, *Rh.krasnovi*, *Rh.incisa*, *Rh.densimaculata* sp. n., *Rh.maculata* sp. n., *Rh.monticola*, and *Rh.saussurei*, of which, eight morphospecies were recovered as single MOTU under both methods; however, there were wide discrepancies of MOTU in the remaining six morphospecies (Figure 1), for example, seven MOTUs in ABGD and four MOTUs in GMYCfor *Rh.nigrovittata*, and seven MOTUs in ABGD and five MOTUs in GMYC for *Rh.melancholica*.

We observed the largest mean K2P intramorphospecies genetic distance was 5% (*Rh.marginata*). The interspecific genetic distance of *Rhabdoblatta* ranged from 8.2 to 19.8% (Table 2).

**Table 2.** Pairwise genetic divergence and the variance of the underlying distribution of distances calculated by using K2P model and bootstrap method respectively using cytochrome oxidase subunit I (COI) gene sequences in MEGA. Bold text denotes the variance of the underlying distribution of distances and black denotes pairwise genetic divergence.

Species	1	2	3	4	5	6	7	8	9	10	11	12	13	14	15	16	17	18
* Rh. monticola *		**0.011**	**0.016**	**0.015**	**0.016**	**0.016**	**0.014**	**0.014**	**0.015**	**0.015**	**0.014**	**0.015**	**0.015**	**0.016**	**0.015**	**0.014**	**0.017**	**0.017**
*Rh.ecarinata* sp. n.	0.097		**0.015**	**0.015**	**0.016**	**0.017**	**0.014**	**0.013**	**0.016**	**0.016**	**0.014**	**0.015**	**0.016**	**0.017**	**0.015**	**0.014**	**0.016**	**0.018**
* Rh. atra *	0.178	0.147		**0.013**	**0.015**	**0.015**	**0.014**	**0.013**	**0.014**	**0.013**	**0.015**	**0.014**	**0.014**	**0.015**	**0.014**	**0.015**	**0.015**	**0.017**
* Rh. rattanakiriensis *	0.172	0.166	0.131		**0.015**	**0.015**	**0.014**	**0.012**	**0.013**	**0.014**	**0.015**	**0.014**	**0.015**	**0.014**	**0.013**	**0.015**	**0.015**	**0.016**
* Rh. elegans *	0.183	0.180	0.151	0.163		**0.010**	**0.013**	**0.014**	**0.015**	**0.014**	**0.015**	**0.015**	**0.015**	**0.017**	**0.015**	**0.016**	**0.014**	**0.014**
* Rh. nigrovittata *	0.198	0.191	0.172	0.162	0.082		**0.014**	**0.014**	**0.015**	**0.015**	**0.014**	**0.016**	**0.016**	**0.017**	**0.015**	**0.016**	**0.014**	**0.013**
* Rh. marginata *	0.173	0.151	0.145	0.162	0.150	0.164		**0.013**	**0.014**	**0.013**	**0.014**	**0.014**	**0.015**	**0.015**	**0.014**	**0.014**	**0.014**	**0.015**
* Rh. sinuata *	0.176	0.138	0.132	0.127	0.149	0.146	0.146		**0.013**	**0.013**	**0.014**	**0.014**	**0.013**	**0.015**	**0.013**	**0.014**	**0.014**	**0.016**
*Rh.similsinuata* sp. n.	0.164	0.165	0.138	0.118	0.144	0.153	0.157	0.127		**0.014**	**0.015**	**0.014**	**0.015**	**0.016**	**0.013**	**0.014**	**0.015**	**0.015**
* Rh. incisa *	0.169	0.162	0.130	0.153	0.143	0.164	0.151	0.130	0.137		**0.014**	**0.015**	**0.010**	**0.015**	**0.012**	**0.015**	**0.016**	**0.014**
*Rh.densimaculata* sp. n.	0.166	0.134	0.148	0.166	0.161	0.159	0.157	0.145	0.156	0.139		**0.015**	**0.015**	**0.015**	**0.014**	**0.015**	**0.016**	**0.015**
* Rh. mascifera *	0.187	0.163	0.127	0.143	0.151	0.167	0.164	0.136	0.131	0.152	0.160		**0.015**	**0.016**	**0.015**	**0.015**	**0.015**	**0.018**
* Rh. krasnovi *	0.172	0.160	0.152	0.160	0.163	0.171	0.171	0.135	0.136	0.085	0.149	0.143		**0.015**	**0.012**	**0.016**	**0.016**	**0.017**
* Rh. bicolor *	0.169	0.173	0.165	0.157	0.171	0.179	0.159	0.154	0.165	0.142	0.152	0.166	0.147		**0.010**	**0.017**	**0.016**	**0.018**
* Rh. melancholica *	0.181	0.164	0.165	0.158	0.166	0.173	0.166	0.158	0.144	0.123	0.154	0.164	0.126	0.100		**0.016**	**0.015**	**0.015**
* Rh. saussurei *	0.140	0.111	0.141	0.169	0.168	0.177	0.152	0.161	0.149	0.160	0.140	0.173	0.164	0.178	0.182		**0.016**	**0.017**
*Rh.maculata* sp. n.	0.183	0.169	0.162	0.167	0.135	0.143	0.157	0.151	0.161	0.159	0.157	0.158	0.161	0.164	0.165	0.178		**0.015**
* Rh. simulans *	0.189	0.191	0.170	0.174	0.117	0.118	0.170	0.167	0.145	0.149	0.162	0.179	0.168	0.181	0.166	0.183	0.133	

### Establishment of six new species

On the basis of morphological characters combined with the molecular data, we were able to identify 20 *Rhabdoblatta* species including six new species among the 504 samples that we examined, i.e., *Rh.similsinuata* sp. n., *Rh.densimaculata* sp. n., *Rh.gyroflexa* sp. n., *Rh.chaulformis* sp. n., *Rh.maculata* sp. n., and *Rh.ecarinata* sp. n.

We attempted to assign 20 *Rhabdoblatta* species into three species groups suggested by Anisyutkin (2003) mainly based on the shape of the apical sclerite of L2D and sclerite L3 hook. Finally we found that ten *Rhabdoblatta* species (*Rh.similsinuata* sp. n., *Rh.densimaculata* sp. n., *Rh.gyroflexa* sp. n., *Rh.marginata*, *Rh.sinuata*, *Rh.incisa*, *Rh.krasnovi*, *Rh.melancholica*, *Rh.bicolor* and *Rh.saussurei*) could not be assigned into any one of the three species groups only using the morphological data listed above. So we didn’t adopt the taxonomic system of species groups in this study.

### Diagnosis of the genus

Vertex slightly exposed; pronotum subelliptical and the widest part in the middle, anterior and lateral margins rounded, middle of hind margin convex; tegmina and wings of male fully developed extending well beyond the end of the abdomen, the apex of the tegmina arc-shaped; anteroventral margin of front femur type B; the metatarsus of hind leg equal length to sum of left tarsi, inner margin with two rows of small spines; the pretarsus with arolium, claws symmetrical and unspecialized; the shape of subgenital plate, apical sclerite of L2D and sclerite L3 hook variously.

**Remarks.** Male genitalia of the species *Rh.similsinuata* sp. n. is very similar to *Rh.sinuata* Bey-Bienko, 1958 and other characters match with generic diagnosis. However, the species shows sexual dimorphism, in which the male macropterous and the female brachypterous.

#### Rhabdoblatta similsinuata sp. n.

Taxon classificationAnimaliaBlattodeaBlaberidae

http://zoobank.org/0B01CA97-0236-45C3-9C03-A09B459A3CB1

Figure 2A–P

##### Diagnosis.

This species is similar to *Rh.sinuata* Bey-Bienko, 1958 in the male genitalia, only with minor differences as follows: hind margin of subgenital plate with an inverted V-shaped concavity at middle, and left lobe slightly processed (with an inverted U-shaped concavity in the middle and left lobe not processed). But this species can easily be differentiated from *Rh.sinuata* in the following characteristics: 1) existence of sexual dimorphism：male macropterous, but female brachypterous (tegmina and wings of male and female fully developed extending well beyond the end of the abdomen in the latter); 2) abdominal sterna with obviously longitudinal bands in the middle (bands absent in the latter).

##### Measurements (mm).

Male, pronotum: length × width 4.3–4.7 × 6.1–6.5, tegmen length: 25.2–25.6, overall length: 29.5–30.3; female, pronotum: length × width 5.2–5.5 × 7.6–8.0, tegmen length: 12.6–13.2, overall length: 21.9–22.4.

##### Description.

**Figure 2.**
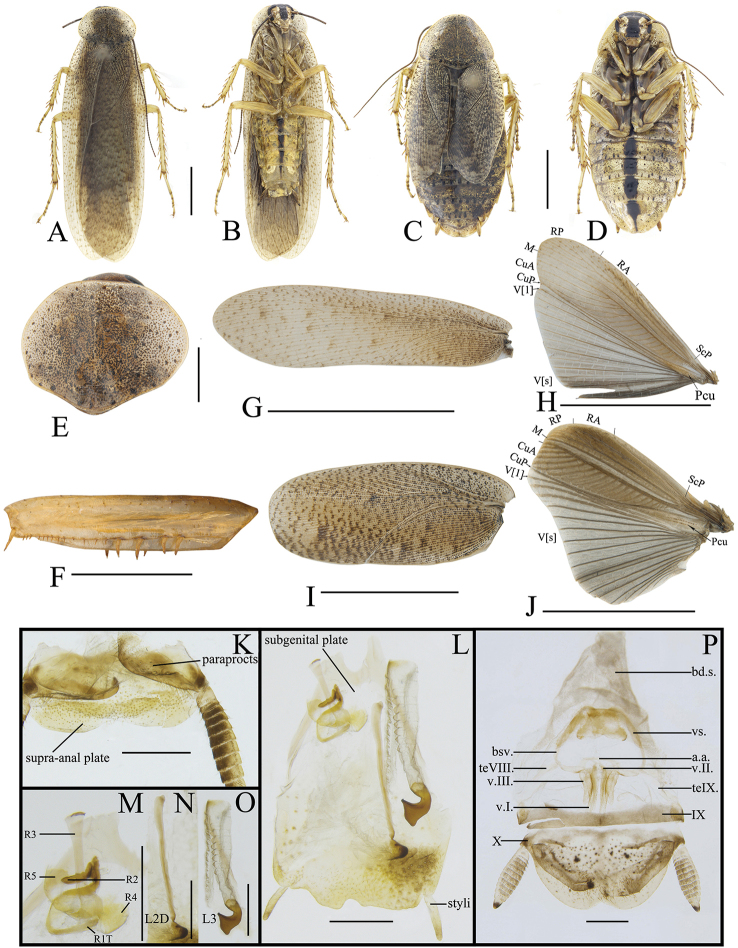
**A, B, E–H, K–O***Rhabdoblattasimilsinuata* sp. n., male **C, D, I, J, P** female. **A** Holotype, dorsal view **B** holotype, ventral view **C** paratype, dorsal view **D** paratype, ventral view **E** pronotum, dorsal view **F** front femur, ventral view **G** tegmen **H** wings **I** tegmen **J** wings **K** supra-anal plate, dorsal view **L** subgenital plate, ventral view **M** left phallomere, ventral view **N** median phallomere, ventral view **O** right phallomere, ventral view **P** female genitalia. Scale bars: 1.0 cm (**G, H**); 5.0 mm (**A–D, I, J**); 1.0 mm (**E, F, K–P**).

**Male.** Body pale yellow (Figure 2A). Eyes blackish brown. Ocelli yellowish white. Antennae dark brown. Vertex, frons and basal of clypeus dark brown, the other part yellow (Figure 2B). Pronotum yellow, with many near round small or a few big black spots on the surface (Figure 2E). Tegmina pale yellow, covered with spots similar to those on pronotum, R and M very close to each other basally (Figure 2G). Wings with costal field, radial field and mediocubital field pale yellow and anal field pale gray, whose veins brown (Figure 2H). Legs yellow. The middle of 3^rd^–6^th^ abdominal sterna with dark brown longitudinal bands forming an inverted triangle, with dispersedly brown spots on the surface of the segments. Cerci brown, apical segment blackish brown (Figure 2B).

Vertex slightly exposed (Figure 2B). Distance between eyes slightly longer than interocular space (Figure 2B). Pronotum subelliptical, the widest part in the middle, anterior and lateral margins rounded, middle of hind margin convex (Figure 2E). Tegmina and wings fully developed extending well beyond the end of the abdomen, the apex of the tegmina arc-shaped and veins distinct (Figure 2A, B, G, H). Anteroventral margin of front femur type B_1_. The inner margin of the metatarsus of hind leg with two rows small spines. Tarsal pulvilli present on the apex of 1^st^-4^th^ tarsomeres, small and spiked, 1^st^–3^rd^ with spines around. The pretarsus with arolium, claws symmetrical and unspecialized (Figure 2B, F).

**Male genitalia.** Supra-anal plate symmetrical, subtrapezoid, the middle of the hind margin concave. Right and left paraprocts unsymmetrical, the right with a big, finger-shaped bulge, the end bent (Figure 2K). Subgenital plate with distal part unsymmetrical, with an inverted V-shaped concave in the middle. The base of the inner plate bifurcated and symmetrical. Styli long and flat, whose length approximately 1/3 of interstyli space (Figure 2L). Left phallomere with sclerite R1T apex nearly rectangle, end of R2 rounded, R3 and R5 interlinked, the base of R3 turned over, and without bifurcation at apex, R4 nearly rectangular and existing independently (Figure 2M). The basal sclerite of L2D slender and rod-shaped, with base slightly intumescent; apical sclerite short and small, the surface on the apical membrane with fine bristles, cap-shaped (Figure 2N). Sclerite L3 long, hook deeply bent and with semicircular carina, margin smooth and with a process; inner margin with tooth-shaped convexity at apex (Figure 2O).

**Female.** Female brachypterous. Tegmina and wings extending to hind margin of 5^th^ abdominal tergum. Cerci yellow, apical segment blackish brown. Abdominal sterna with longitudinal broad band in the middle, and finger-like spots along the hind margin, and brown spots dispersed on the surface of the segments (Figure 2C, D, I, J).

**Female genitalia.** Weakly sclerotized. Ovipositor back to brood sac. Tergal process of the eighth abdominal tergite obviously vestigial, getting narrower from the base to the end, length approximately half of tergal process of the ninth abdominal tergite. Tergal process of the ninth abdominal tergite slightly wider, connected to the ninth tergum. First valves of ovipositor with narrow and fine membrane at the apex, inner margin with clearly fine and long bristles. Second valves of ovipositor tube-shaped, completely covered by the first valves of ovipositor. Third valves of ovipositor slightly wider, length shorter than the first valves of ovipositor. Gonangulum and sclerotized lobes of the second and third pairs of valves absent. Anterior arch of second valvifer slender. Basivalvula with semicircular arms, the middle sclerite incompletely separated, semicircular. Vestibular sclerite weakly sclerotized, the middle sclerite slightly membranous. Transverse sclerotized plate absent. Brood sac membranous and without sclerotized section (Figure 2P).

##### Remarks.

The status of *Rhabdoblattasimilsinuata* sp. n. is proven to be valid according to our morphological and molecular data (the interspecific genetic distance between this species and *Rh.sinuata*: 0.120).

##### Etymology.

Latin word *similis* means similar, referring to the male genitalia being similar to *Rh.sinuata* Bey-Bienko, 1958.

##### Type material.

*Holotype*: male, Yunnan Prov., Xinping County, Ailao Mountain, Yaonan Village, 11–13-V-2016, Lu Qiu & Zhi-wei Qiu leg. *Paratype*: 11 males and 17 females, same data as holotype; 1 female, Yunnan Prov., Xinping County, Ailao Mountain, Yaonan Village, 23-V-2018, Lu Qiu, Wen-bo Deng & Zhi-wei Dong leg. (all in SWU).

##### Distribution.

China (Yunnan).

#### Rhabdoblatta densimaculata sp. n.

Taxon classificationAnimaliaBlattodeaBlaberidae

http://zoobank.org/0DA63989-4C49-46B1-A376-C0D1505942F1

Figure 3A–P

##### Diagnosis.

This species is similar to *Rh.incisa* Bey-Bienko, 1969 in the spots of tegmina. But this species can easily be differentiated from *Rh.incisa* in the following characteristics: 1) body brown, but dark brown in the latter; 2) pronotum yellow, black small spots dispersed on the surface (pronotum dark brown, lateral borders with pale spots in the latter); and 3) sclerite L3 long, hook short and small, and without carina (hook deeply bent and with carina in the latter).

##### Measurements (mm).

Male, pronotum: length × width 6.1–6.5 × 7.3–7.8, tegmen length: 31.8–32.8, overall length: 37.9–39.3; female, pronotum: length × width 7.8 × 9.0–9.3, tegmen length: 31.2–32.3, overall length: 39.0–40.1.

##### Description.

**Figure 3.**
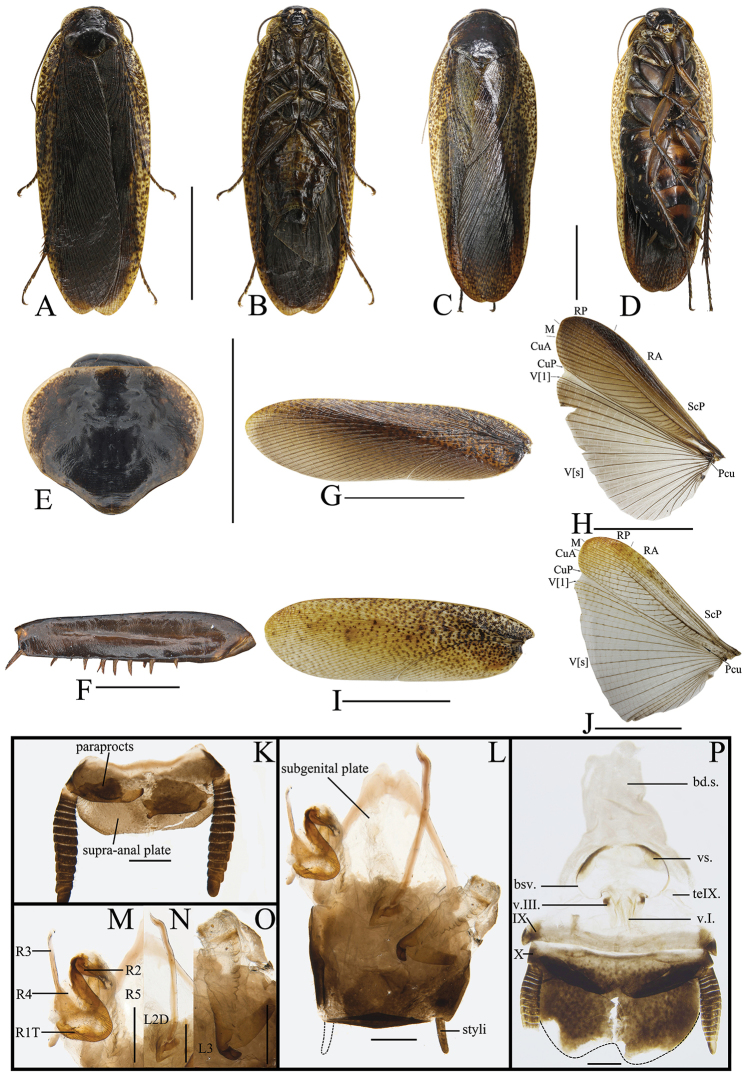
**A, B, E–H, K–O***Rhabdoblattadensimaculata* sp. n., male **C, D, I, J, P** female. **A** Holotype, dorsal view **B** holotype, ventral view **C** paratype, dorsal view **D** paratype, ventral view **E** pronotum, dorsal view **F** front femur, ventral view **G** tegmen **H** wings **I** tegmen **J** wings **K** supra-anal plate, dorsal view **L** subgenital plate, ventral view **M** left phallomere, ventral view **N** median phallomere, ventral view **O** right phallomere, ventral view **P** female genitalia. Scale bars: 1.0 cm (**A–D, G–J**); 5.0 mm (**E**); 1.0 mm (**F, K–P**).

**Male.** Body dark brown (Figure 3A). Vertex, frons, and eyes black. The 1^st^–12^th^ segments of antennae dark brown, the others brown. Ocelli and apex of clypeus yellow. Labrum, labial palpi, and maxillary palpi brown (Figure 3B). Pronotum dark brown, lateral borders with pale spots (Figure 3E). Tegmina dark brown, front borders pale brown, with dark brown spots. Wings with costal field and radial field dark brown, mediocubital field brown and anal field gray, with veins obvious and brown (Figure 3G, H). Legs dark brown. Abdominal sterna yellow, 4^th^–6^th^ segments with dark brown spots. Cerci dark brown (Figure 3B).

Vertex slightly exposed (Figure 3B). Distance between eyes slightly wider than interocular space, the length approximately 2/3 of the space of antennal sockets. The length of third maxillary palpus same as the fifth, both slightly longer than the fourth (Figure 3B). Pronotum subelliptical, the widest part in the middle, anterior and lateral margins rounded, middle of hind margin convex (Figure 3E). Tegmina and wings fully developed extending well beyond the end of the abdomen, the apex of the tegmina arc-shaped and veins distinct (Figure 3A, B, G, H). Anteroventral margin of front femur type B_1_ (Figure 3F). The inner margin of the metatarsus of hind leg with two rows small spines. Tarsal pulvilli present on the apex of 1^st^–4^th^ tarsomeres. The pretarsus with arolium, claws symmetrical and unspecialized (Figure 3B).

**Male genitalia.** Supra-anal plate subtrapezoid, lateral margins arc-shaped. Right and left paraprocts unsymmetrical, shape similar to other members in the genus (Figure 3K). Subgenital plate with hind margin curved upturn. The base of the inner plate bifurcated. Styli flat, whose length ca. 1/3 of interstyli space (Figure 3L). Left phallomere with bristles, end of sclerite R2 rounded, R3 and R5 interlinked, base of R3 turned over and without bifurcation at apex, and R4 weakly sclerotized and existing independently (Figure 3M). L2D slender, basal part sharp and apex straight; apical sclerite nearly rectangle, the membrane with fine bristles, simple cap-shaped (Figure 3N). Sclerite L3 long, hook short and small, outer-lateral margin arc-shaped, smooth and without carina; inner margin with a tooth-shaped convexity at apex (Figure 3O).

**Female.** Female similar to male but slightly bigger. Ocelli, apex of clypeus and labrum yellow. Color of the body and spots similar to male (Figure 3C, D, I, J).

**Female genitalia.** Weakly sclerotized. Ovipositor back to brood sac. Tergal process of the eighth and ninth abdominal tergite obviously vestigial, membranous. First valves of ovipositor with apex membranous, inner margin with fine bristles. Second valves of ovipositor fine, tube-shaped, completely covered by the first valves of ovipositor. Third valves of ovipositor slightly wider and flat, length shorter than the first valves of ovipositor. Gonangulum and sclerotized lobes of the second and third pairs of valves absent. Anterior arch of second valvifer obviously vestigial. Basivalvula weakly sclerotized and with semicircular arms, the mid sclerite separate. Vestibular sclerite wide and weakly sclerotized. Transverse sclerotized plate disappeared. Brood sac membranous and without sclerotized section (Figure 3P).

##### Etymology.

This species name is derived from the Latin words *densus* and *maculatus*, referring to the tegmina having dense spots.

##### Type material.

*Holotype*: male, Sichuan Prov., Ya’an City, Yingjing County, Longcanggou National Forest Park, 19-VI-2016, Jian-yue Qiu leg. *Paratype*: 6 males and 2 females, same data as holotype; 2 males, Sichuan Prov., Chengdu City, Congzhou, Jiguan Mountain, Shaoyaogou, 28-V-2016, Fu-ming Shi leg.; 1 male, Sichuan Prov., Chengdu City, Congzhou, Jiguanshan Township, Anzihe Nature Reserve, 1500m, VI-2015, by light trap, Chao Zhou leg.; 1 male, Sichuan Prov., Chengdu City, Congzhou, Jiguanshan Township, Anzihe Nature Reserve, 1450 m, 2-VI-2016, by light trap, Chao Zhou leg.; 1 female, Yunnan Prov., Dali City, Yunlong County, Mt. Zhiben, 2250 m, 1-VI-1981, Su-bai Liao leg.; 1 female, Yunnan Prov., Xinping County, Ailao Mountain, Yaonan Village, 11-V-2016, Lu Qiu & Zhi-wei Qiu leg.; 1 male, Yunnan Prov., Tengchong City, Diantan Town, 3–15-VII-2016, light trap, Gui-qiang Huang leg.; 4 females, Yunnan Prov., Gongshan County, Dulongjiang Township, 1400 m, 22–28-VII-2015, Chao Wu leg.; 2 females, Xizang Auto. Regi., Medog County, 80k (Bolonggong), 20–24-VII-2012, Chao Wu leg.; 1 female, Xizang Auto. Regi., Medog County, Beibeng Township, Gelin Village, 12-VII-2016, Hao Xu et Jian-yue Qiu leg. (all in SWU).

##### Distribution.

China (Sichuan, Yunnan, Xizang).

#### Rhabdoblatta gyroflexa sp. n.

Taxon classificationAnimaliaBlattodeaBlaberidae

http://zoobank.org/4000A036-211B-4D8E-B02B-FEE2C5C97F21

Figure 4A–K

##### Diagnosis.

This species is similar to *Rh.elegans* Anisyutkin, 2000 in body color, but can be differentiated by the following characters: 1) pronotum reddish yellow with a subtrapezoid dark brown marking at disc (pronotum reddish brown without any marking at disc in the latter); 2) body large (the latter with body medium); and 3) stripes absent in the abdomen (long and black stripes at the hind margin of each segment of the abdominal sterna in the latter); 4) apical part of sclerite R3 of left phallomere turned over (none in the latter).

##### Measurements (mm).

Male, pronotum: length × width 7.5–8.0 × 10.0–10.5, tegmen length: 39.5–42.0, overall length: 44.5–46.0.

##### Description.

**Figure 4.**
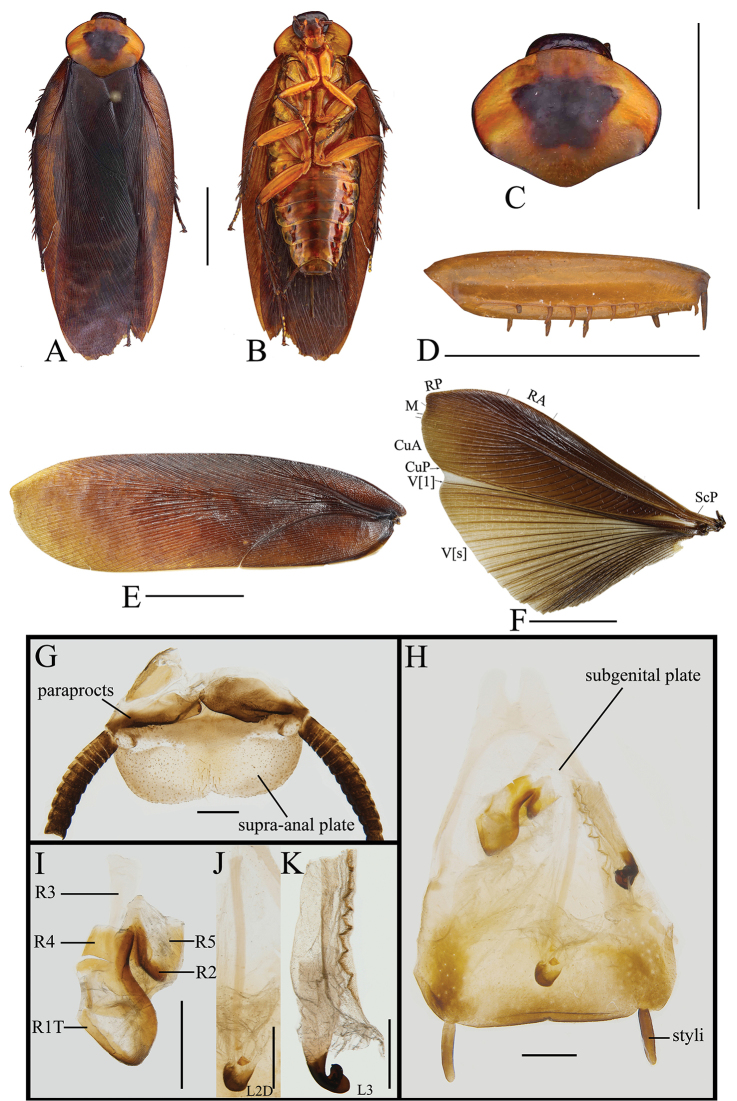
**A–H***Rhabdoblattagyroflexa* sp. n., male. **A** Holotype, dorsal view **B** holotype, ventral view **C** pronotum, dorsal view **D** front femur, ventral view **E** tegmen **F** wings **G** supra-anal plate, dorsal view **H** subgenital plate, ventral view **I** left phallomere, ventral view **J** median phallomere, ventral view **K** right phallomere, ventral view. Scale bars: 1.0 cm (**A–C, E, F**); 5.0 mm (**D**); 1.0 mm (**G–K**).

**Male.** Body reddish brown (Figure 4A). Vertex, antennae and eyes dark brown. Ocelli yellowish brown. Frons reddish brown, but space between ocelli dark brown. Clypeus yellowish brown. Mandible and labrum yellowish brown. The first and second of maxillary palpi yellowish brown, the others dark brown (Figure 4B). Pronotum reddish yellow, with a subtrapezoid dark brown marking at disc (Figure 4C). Tegmina with mediocubital field brown, the other field reddish brown. Wings with costal field, radial field, and mediocubital field brown and anal field yellowish brown with veins brown (Figure 4E, F). Coxa, trochanter and femur yellowish brown; the distal part of femur, tibia, tarsomere dark brown. Abdomen brown, abdominal sterna with blackish brown spots along the lateral margins, and with yellow stripes at the lateral and hind margins of the segments. Cerci dark brown (Figure 4B).

Vertex slightly exposed (Figure 4B). Eyes wide, the hind margin extending to the base of the mandible (Figure 4B). Pronotum subelliptical, the widest part in the middle, anterior and lateral margins rounded, middle of hind margin distinctly convex (Figure 4C). Tegmina and wings fully developed extending well beyond the end of the abdomen, the apex of the tegmina and wings with a small convexity and veins clearly (Figure 4A, B, E, F). Anteroventral margin of front femur type B_2_ (Figure 4D). The metatarsus of hind leg equal length to sum of left tarsi, inner margin with two rows of small spines. Tarsal pulvilli present on the apex of 1^st^–4^th^ tarsomeres. The pretarsus with arolium, claws symmetrical and unspecialized (Figure 4B).

**Male genitalia.** Supra-anal plate nearly semicircular, the middle of the hind margin slightly concave. Right and left paraprocts unsymmetrical, shape similar to other members in the genus (Figure 4G). Subgenital plate with hind margin nearly straight. The base of the inner plate bifurcated. Styli long and flat, whose length ca. 1/3 of interstyli space (Figure 4H). Left phallomere with sclerite R1T apex near square, end of R2 rounded, R3 and R5 interlinked, the base of R3 turnover and without bifurcation at apex, R4 nearly rectangle and existing independently (Figure 4I). The basal sclerite of L2D slender and rod-shaped, apical sclerite short and small; the surface of the apical membrane with fine bristles, cap-shaped (Figure 4J). Sclerite L3 hook deeply bent and with semicircular carina with margin smooth; inner margin with groove and a tooth-shaped convexity at apex (Figure 4K).

##### Female.

Female unknown.

##### Etymology.

This species epithet is derived from the Latin words *gyroflexus*, referring to the yellowish brown marking on the pronotum.

##### Type material.

*Holotype*: male, Guangxi Prov., Congzuo City, Pingxiang, 8-V-1963, Ji-kun Yang leg. *Paratype*: 1 male, same data as holotype; 1 male, Guangxi Prov., Congzuo City, Pingxiang, 8-V-1963, Si-kong Liu leg. (all in SWU).

##### Distribution.

China (Guangxi).

#### Rhabdoblatta chaulformis sp. n.

Taxon classificationAnimaliaBlattodeaBlaberidae

http://zoobank.org/6CD7D802-2BEF-458B-AE76-0BDE9C983B7B

Figure 5A–K

##### Diagnosis.

Sclerite L2D is strongly sclerotized with a exclamation-shaped process, it is the unique diagnosis of this species.

##### Measurements (mm).

Male, head: length × width 3.5 × 3.0, pronotum: length × width 5.5 × 7.0, tegmen length: 28, overall length: 30.0–31.0.

##### Description.

**Figure 5.**
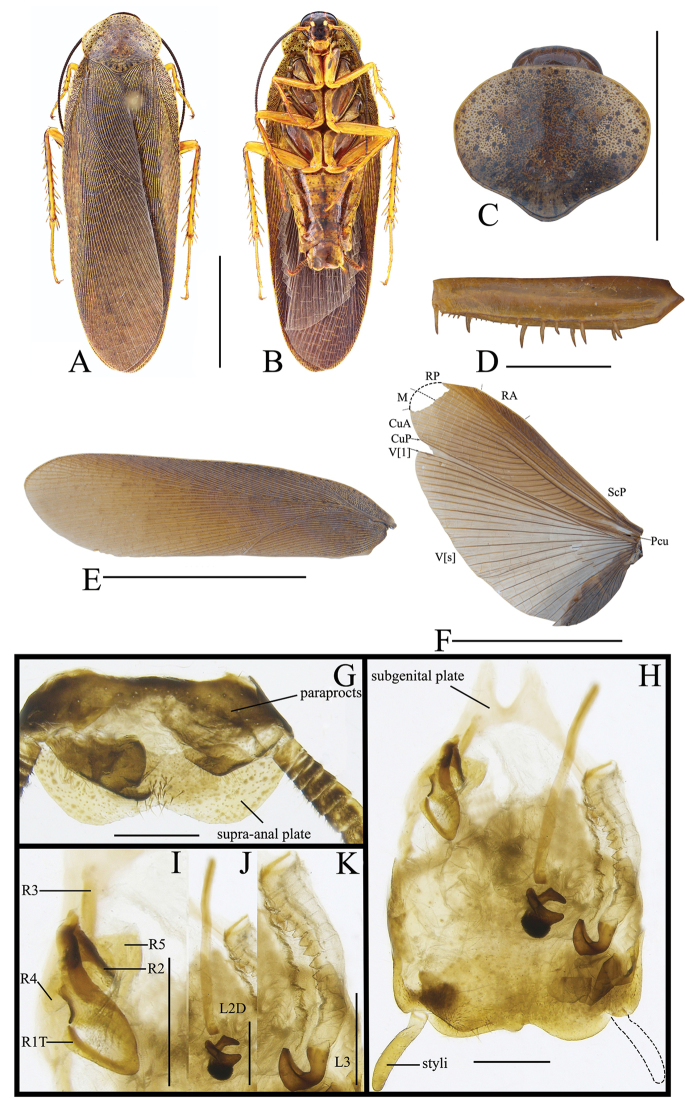
**A–H***Rhabdoblattachaulformis* sp. n., male. **A** Holotype, dorsal view **B** holotype, ventral view **C** pronotum, dorsal view **D** front femur, ventral view **E** tegmen **F** wings **G** supra-anal plate, dorsal view **H** subgenital plate, ventral view **I** left phallomere, ventral view **J** median phallomere, ventral view **K** right phallomere, ventral view. Scale bars: 1.0 cm (**A, B, E, F**); 5.0 mm (**C**); 1.0 mm (**D, G–K**).

**Male.** Body yellowish brown (Figure 5A). Vertex dark brown. Eyes dark brown, border yellow. Ocelli yellow. Scape of antennae yellowish brown, the other dark brown. Frons dark brown. The base of clypeus dark brown, remaining part yellow. Mandible and labrum yellow. Maxillary palpi with the fifth brown, the others yellow (Figure 5B). Pronotum yellow, disc brown, with dark brown spots on the surface, with longitudinal short stripes along hind margin (Figure 5C). Tegmina brown, veins yellow. Wings with costal field, radial field, and mediocubital field yellowish brown and anal field pale brown, veins obvious and brown (Figure 5E, F). Legs brown. Abdominal terga dark brown, sterna yellow and with scattered blackish brown spots. Cerci dark brown (Figure 5B).

Vertex slightly exposed (Figure 5B). Distance between eyes slightly wider than interocular width, the length ca. 2/3 of the space between antennal sockets (Figure 5B). Pronotum subelliptical, the anterior and lateral margins rounded, middle of hind margin distinctly convex (Figure 5C). Tegmina and wings fully developed extending well beyond the end of the abdomen, the apex of the tegmina and wings slightly protruding and veins distinct (Figure 5A, B, E, F). Anteroventral margin of front femur type B_1_ (Figure 5D). The metatarsus of hind leg longer than the sum of left tarsi, the inner margin with two rows of small spines. Tarsal pulvilli present on the apex of 1^st^–4^th^ tarsomeres, small and spiked. Arolium present, claws symmetrical and unspecialized (Figure 5B).

**Male genitalia.** Supra-anal plate nearly semicircle, symmetrical, the middle of the hind margin slightly concave. Right and left paraprocts unsymmetrical, shape similar to other members in the genus (Figure 5G). Subgenital plate with hind margin unsymmetrical, slightly M-shaped margin. The base of the inner plate bifurcated. Styli long, whose length ca. 1/2 of interstyli space (Figure 5H). Left phallomere with sclerite R1T with bristles, end of R2 rounded, R3 and R5 interlinked, R4 weakly sclerotized (Figure 5I). The basal sclerite of L2D slender and rod-shaped, almost straight, apical sclerite irregular and with an exclamation-shaped process; the surface on the apical membrane with fine bristles, cap-shaped (Figure 5J). Sclerite L3 slender and hook deeply bent, and with semicircular carina; inner margin with groove and a tooth-shaped convexity at apex (Figure 5K).

**Female.** Female unknown.

##### Etymology.

This species epithet is derived from the Latin words *chaul* and *formis*, referring to L2D with a exclamation-shaped process.

##### Type material.

*Holotype*: male, Chongqing City, Wanzhou Dist, Wangerbao Nature Reserve, 1700 m, 2-V-2007, Wei-wei Zhang leg. *Paratype*: 1 male, same data as holotype (all in SWU).

##### Distribution.

China (Chongqing).

#### Rhabdoblatta maculata sp. n.

Taxon classificationAnimaliaBlattodeaBlaberidae

http://zoobank.org/AAC32271-2194-4D0B-8ECE-AB62FDCA275C

Figure 6A–K

##### Diagnosis.

This species is similar to *Rhabdoblattaomei* Bey-Bienko, 1958, but can be differentiated by the following characters: 1) body wider with darker spots on pronotum and tegmina (body narrower with pale spots on pronotum and tegmina in the latter); 2) the hind margin of subgenital plate slightly concave and nearly symmetrical (the latter with the hind margin obviously concave and asymmetrical); and 3) outer-lateral margin apex of L3 with carina blunt and rounded (sharp and acute in the latter).

##### Measurements (mm).

Male, head: length × width 4.8 × 5.0, pronotum: length × width 7.8 × 10.0, tegmen length: 39.1, overall length: 41.7–43.5.

##### Description.

**Figure 6.**
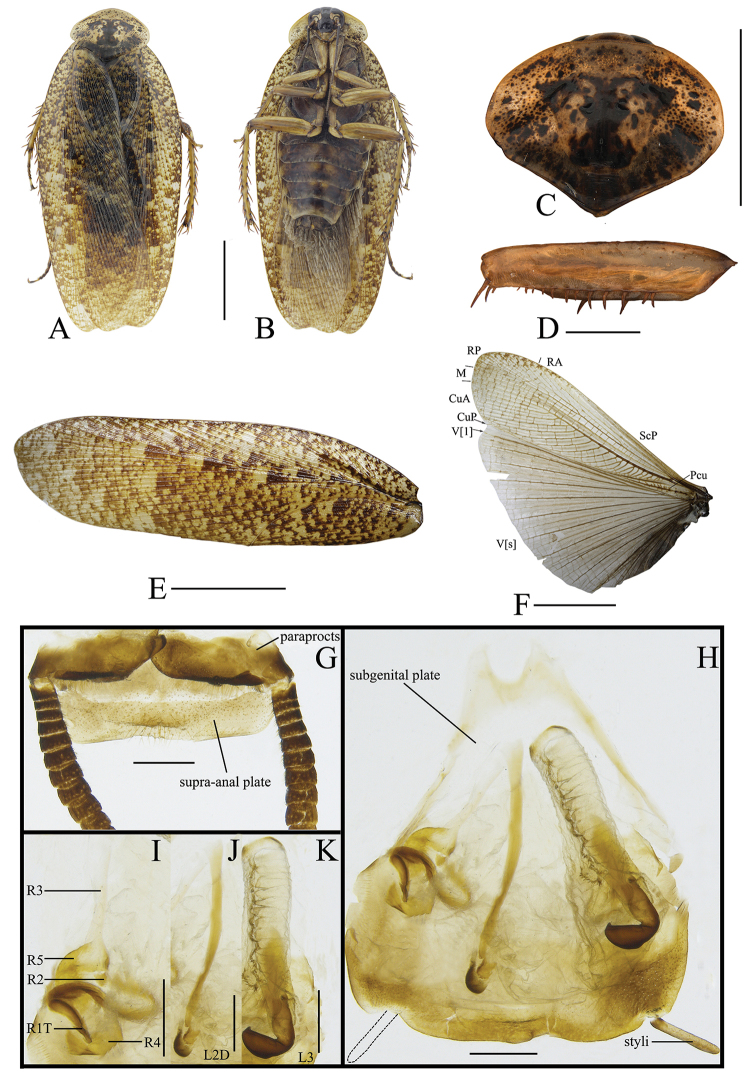
**A–H***Rhabdoblattamaculata* sp. n., male. **A** Holotype, dorsal view **B** holotype, ventral view **C** pronotum, dorsal view **D** front femur, ventral view **E** tegmen **F** wings **G** supra-anal plate, dorsal view **H** subgenital plate, ventral view **I** left phallomere, ventral view **J** median phallomere, ventral view **K** right phallomere, ventral view. Scale bars: 1.0 cm (**A, B, E, F**); 5.0 mm (**C**); 1.0 mm (**D, G–K**).

**Male.** Body yellow (Figure 6A). Vertex, eyes, and frons black. The apex of clypeus yellow, the remaining black. Ocelli yellow. Scape of antennae brown, the 2^nd^–15^th^ segments dark brown, other segments pale brown. Mandible and labrum yellow. Maxillary palpi brown (Figure 6B). Pronotum yellow, with an irregular and symmetrical dark brown marking at disc, and with messy and dense brown spots on the border, posterior margin with longitudinal short stripes (Figure 6C). Tegmina yellow, with numerous scattered dark brown or brown spots. Wings pale gray, veins yellowish brown (Figure 6E, F). Legs reddish brown. Abdominal sterna brown. Cerci dark brown (Figure 6B).

Vertex slightly exposed (Figure 6B). Distance between eyes slightly narrower than interocular width, length ca. 1/2 of the space of antennal socket (Figure 6B). Pronotum subelliptical, the anterior and lateral margins rounded, middle of hind margin distinctly convex (Figure 6C). Tegmina and wings fully developed extending well beyond the end of the abdomen, the apex of the tegmina with a convex and wings blunt and rounded (Figure 6A, B, E, F). Anteroventral margin of front femur type B_2_ (Figure 6D). The metatarsus of hind leg equal length to sum of left tarsi, the inner margin with two rows of small spines. Tarsal pulvilli present on the 1^st^–4^th^ of the tarsomere apex, with 1–2 spines. The pretarsus with arolium, claws symmetrical and unspecialized (Figure 6B).

**Male genitalia.** Supra-anal plate nearly semicircular, lateral margins rounded, the middle of the hind margin slightly concave. Right and left paraprocts unsymmetrical, shape similar to other members in this genus (Figure 6G). Subgenital plate with hind margin nearly symmetrical, right part with concavity. The base of the inner plate bifurcated. Styli flat, length ca. 1/3 of interstyli space (Figure 6H). The apex of the sclerite R1T peaked, end of R2 rounded, R3 and R5 interlinked; R4 existing independently (Figure 6I). The basal sclerite of L2D slender and rod-shaped; apical sclerite short and small, the surface on the apical membrane with fine bristles, cap-shaped (Figure 6J). Sclerite L3 long, with blunt and rounded carina; inner margin with groove and a tooth-shaped convexity at apex (Figure 6K).

**Female.** Female unknown.

##### Etymology.

This species epithet is derived from the Latin word *maculatus*, referring to the tegmina having clear spots.

##### Type material.

*Holotype*: male, Guizhou Prov., Leigongshan Mountain, 6-VI-2013, Gui-qiang Huang & Xiang-xiang Zhang leg. *Paratype*: 1 male, same data as holotype; 1 male, Guizhou Prov., Leigongshan Mountain, 29-VI-1988, Min-sheng Wang leg. (all in SWU).

##### Distribution.

China (Guizhou).

#### Rhabdoblatta ecarinata sp. n.

Taxon classificationAnimaliaBlattodeaBlaberidae

http://zoobank.org/FD060B05-39AA-4C2A-833A-BAA77DD5DAA1

Figure 7A–T

Rhabdoblatta carinata Liu et al., 2017: 78 (nomen nudum).

##### Diagnosis.

The outer-lateral margin of the sclerite L3 hook without carina, and it is similar to *Rhabdoblattamonticola* (Kirby, 1903), but subcosta of *Rh.monticola* is white (Figure 8A), and this species is yellowish brown.

##### Measurements (mm).

Male, pronotum: length × width 5.0–6.0 × 7.5–8.0, tegmen length: 22.0–24.0, overall length: 26.0–28.0; female, pronotum: length × width 7.8–8.0 × 8.4–9.0, tegmen length: 31.0–32.0, overall length: 37.0–38.0.

##### Description.

**Figure 7.**
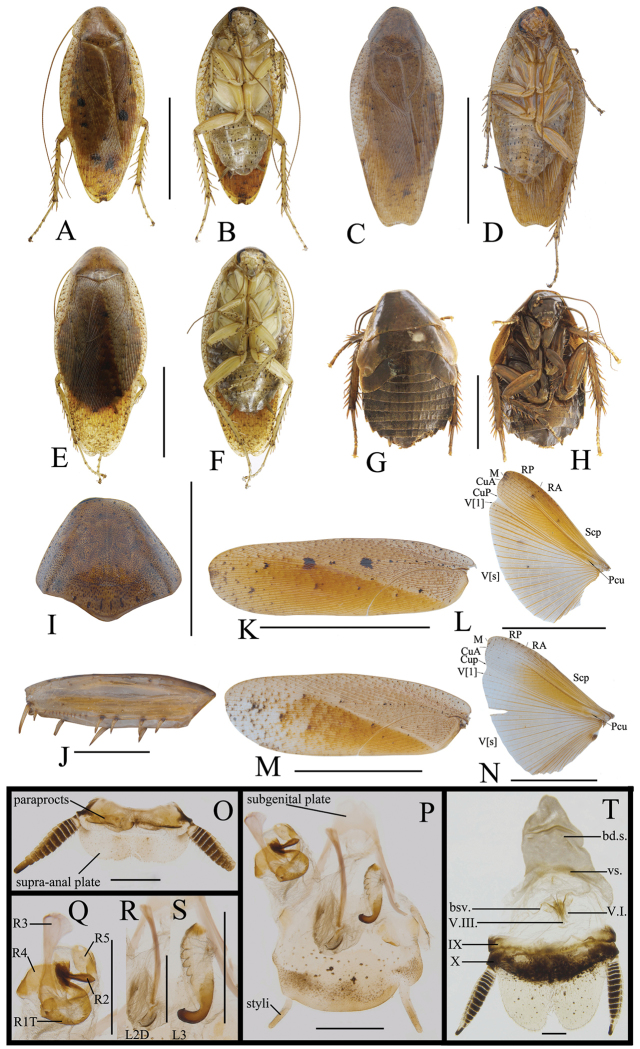
**A–D, I–L, O–S***Rhabdoblattaecarinata* sp. n., male **E, F, M, N, T** female **G, H** nymph. **A** Paratype, dorsal view **B** paratype, ventral view **C** holotype, dorsal view **D** holotype, ventral view **E** paratype, dorsal view **F** paratype, ventral view **G** nymph, dorsal view **H** nymph, ventral view **I** pronotum, dorsal view **J** front femur, ventral view **K** tegmen **L** wings **M** tegmen **N** wings **O** supra-anal plate, dorsal view **P** subgenital plate, ventral view **Q** left phallomere, ventral view **R** median phallomere, ventral view **S** right phallomere, ventral view **T** female genitalia. Scale bars: 1.0 cm (**A–H, K–N**); Scale bars: 5.0 mm (**I**); 1.0 mm (**J, O–T**).

**Male.** Body yellowish brown (Figure 7A). Vertex and apex of frons with scattered brown spots. Eyes black. Ocelli pale yellow. Labrum, labial palpi, and maxillary palpi yellow (Figure 7B). Pronotum yellowish brown, with many near round small or a few big brown spots on the surface and longitudinal short stripes along hind margin (Figure 7I). Tegmina yellowish brown, with several large dark brown spots on the surface or not. Wings with costal field, radial field, and mediocubital field yellowish brown, and anal field gray, with veins obvious and yellow (Figure 7K, L). Legs yellow. Abdominal sterna yellow, 4^th^–7^th^ segments with dark brown spots. Cerci dark brown (Figure 7B).

Vertex slightly exposed. Distance between eyes slightly wider than interocular space, the length ca. 2/3 of the space of antennal sockets (Figure 7B). Pronotum subelliptical, the widest part in the middle, anterior and lateral margins rounded, middle of hind margin convex (Figure 7I). Tegmina and wings fully developed extending well beyond the end of the abdomen, the apex of the tegmina arc-shaped and veins distinct (Figure 7A–D, K, L). Anteroventral margin of front femur type B_2_ (Figure 7J). The inner margin of the metatarsus of hind leg with two rows small spines. Tarsal pulvilli present on the apex of 1^st^–4^th^ tarsomeres. The pretarsus with arolium, claws symmetrical and unspecialized (Figure 7B).

**Male genitalia.** Supra-anal plate symmetrical, nearly rectangular, the middle of the hind margin with concavity (Figure 7O). Subgenital plate with hind margin unsymmetrical, with a cambered convexity in the middle, the left stylus shorter than the right (Figure 7P). Left phallomere with sclerite R3 intamescent at apex, R4 wide and nearly square (Figure 7Q). The basal sclerite of L2D slender and rod-shaped, base slightly intamescent; apical sclerite small, the surface on the apical membrane with fine bristles (Figure 7R). Sclerite L3 with small hook, outer-lateral margin without carina, inner margin with a tooth-shaped convexity at apex (Figure 7S).

**Female.** Female similar to male but slightly larger (Figure 7E, F, M, N).

**Female genitalia.** Weakly sclerotized. Ovipositor back to brood sac. Tergal process of the eighth and ninth abdominal tergite obviously vestigial. First valves of ovipositor wide, apex membranous. Second valves of ovipositor fine and tube-shaped, completely covered by the first valves of ovipositor. Third valves of ovipositor slightly wider and flat, length shorter than the first valves of ovipositor. Gonangulum and sclerotized lobes of the second and third pairs of valves not obvious. Anterior arch of second valvifer obviously vestigial. Basivalvula with semicircular arms, the mid sclerite with incomplete separation, linked with membrane. Vestibular sclerite membranous, wider than the basivalvula. Transverse sclerotized plate absent. Brood sac membranous and without sclerotized section (Figure 7T).

**Nymph.** Body brown. Spine on the tibia robust. The length of antennae nearly equal to the body’s length (Figure 7G, H).

##### Remarks.

This species was named as *Rhabdoblattacarinata* by Liu et al. (2017) in the book Cockroaches of Southeastern China (page 78). However, no exact deposition of the type specimens was mentioned, although the authors listed three collections in the material and method. Based on Article 16.4.2 of the International Code of Zoological Nomenclature (ICZN 1999), the name *Rhabdoblattacarinata* is not available. Based on the material we examined, males of the species have intraspecific variation, with some individuals having dispersed large dark brown spots on tegmina, while others do not (Figure 7A–D).

##### Etymology.

This species name is derived from the Latin word *ecarinatus*, referring to the outer-lateral margin of the sclerite L3 hook without carina.

##### Type material.

*Holotype*: male, Hainan Prov., Yinggeling Nature Reserve, Nanfa Conservation Station, 650m, 21-IV-2015, Lu Qiu & Qi-kun Bai leg. *Paratype*: 8 males and 5 females, Hainan Prov., Yinggeling Nature Reserve, Nankai Conservation Station, 284–308m, 20-IV-2015, Xin-ran Li & Zhi-wei Qiu leg.; 2 females, Hainan Prov., Lingshui County, Diaoluoshan Mountain, 15-IV-2015, Lu Qiu & Qi-kun Bai leg.; 1 female, 1 male, 1 nymph, Hainan Prov., Lingshui County, Diaoluoshan Mountain, 24-V-2014, Shun-hua Gui & Xin-ran Li leg. (all in SWU).

##### Distribution.

China (Hainan).

### First descriptions of females including female genitalia of the 14 known *Rhabdoblatta* species

The DNA Barcode method allows us to successfully match *Rhabdoblatta* male and female samples, in spite of sexual dimorphism. Therefore, we take this opportunity to describe for the first time the female genitalia of 14 known *Rhabdoblatta* species.

#### Rhabdoblatta monticola

Taxon classificationAnimaliaBlattodeaBlaberidae

(Kirby, 1903)

Figures 8A–D , 12E

Rhabdoblatta monticola , Princis, 1967: 664; Anisyutkin 2003: 542.

##### Measurements (mm).

Female, overall length: 38.3–40.5.

##### Female.

**Figure 8.**
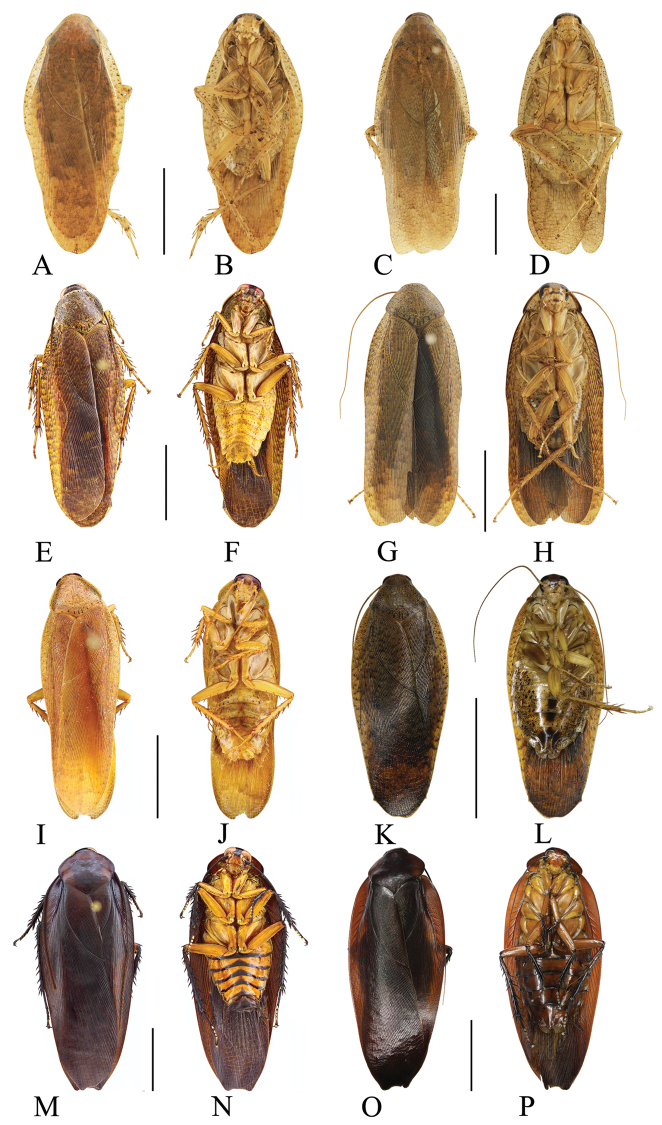
**A–D***Rhabdoblattamonticola* (Kirby, 1903): **A, B** male **C, D** female **E–H***Rhabdoblattaatra* Bey-Bienko, 1970: **E, F** male **G, H** female **I–L***Rhabdoblattarattanakiriensis* Anisyutkin, 1999: **I, J** male **K, L** female **M–P***Rhabdoblattaelegans* Anisyutkin, 2000: **M, N** male **O, P** female. Scale bars: 1.0 cm.

Female similar to male but slightly larger (Figure 8A–D).

##### Female genitalia.

Weakly sclerotized. Ovipositor back to brood sac. Tergal process of the eighth abdominal tergite obviously vestigial, length ca. 1/2 of tergal process of the ninth abdominal tergite. Tergal process of the ninth abdominal tergite and the ninth tergum interlinked. First valves of ovipositor wide, apex membranous, inner margin with long bristles. Second valves of ovipositor fine, tube-shaped, completely covered by the first valves of ovipositor. Third valves of ovipositor slightly wider and flat, length shorter than the first valves of ovipositor. Gonangulum irregular. Sclerotized lobes of the second and third pairs of valves nearly triangular. Anterior arch of second valvifer slender. Basivalvula with semicircular arms, the mid sclerite incompletely separated, linked with membrane. Vestibular sclerite wide, wider than the basivalvula. Transverse sclerotized plate bowknot-shaped. Brood sac membranous and without sclerotized section (Figure 12E).

##### Material examined.

1 male and 7 females, Guangxi Prov., Shangsi County, Shiwandashan National Forest Park, 28-VI-2015, Lu Qiu & Qi-kun Bai leg.; 1 male and 1 female, Guangxi Prov., Jinxiu County, Dayaoshan Nature Reserve, Hekou Reserve Station, 4-VII-2015, Lu Qiu & Qi-kun Bai leg.; 1 female, Guangdong Prov., Zhaoqing City, Dinghushan Forest Park, 1–2-VII-2015, Zhi-wei Qiu & Yong-quan Zhao leg.; 1 male, Guangdong Prov., Zhaoqing City, Fenghuang Town, Jiulonghu Lake, 4-VII-2015, Zhi-wei Qiu & Yong-quan Zhao leg.

##### Distribution.

China (Guangxi, Guangdong); Vietnam.

#### Rhabdoblatta atra

Taxon classificationAnimaliaBlattodeaBlaberidae

Bey-Bienko, 1970

Figures 8E–H , 12F

Rhabdoblatta atra Bey-Bienko, 1970: 364; Anisyutkin 2003: 554.

##### Measurements (mm).

Female, overall length: 29.0–33.0.

##### Female.

Female similar to male but slightly larger. The hind margin of every abdominal sterna with scattered large brown spots (Figure 8E–H).

##### Female genitalia.

Moderately sclerotized. Ovipositor back to brood sac. Tergal process of the eighth abdominal tergite slender, length ca. 1/2 of tergal process of the ninth abdominal tergite. Tergal process of the ninth abdominal tergite weakly sclerotized, linked with the ninth tergum. First valves of ovipositor with base wide, apex narrow, inner margin with obviously long bristles. Second valves of ovipositor tube-shaped, completely covered by the first valves of ovipositor. Third valves of ovipositor slightly wider, length shorter than the first valves of ovipositor. Gonangulum absent. Sclerotized lobes of the second and third pairs of valves not obvious. Anterior arch of second valvifer slender, middle narrow and both sides wide. Basivalvula with semicircular arms, the mid sclerite incompletely separated. Vestibular sclerite membranous, apical sclerite vestigial. Transverse sclerotized plate subelliptical. Brood sac membranous and without sclerotized section (Figure 12F).

##### Material examined.

40 males and 6 females, Guangxi Prov., Jinxiu County, Dayaoshan Nature Reserve, Hekou Reserve Station, 7–8-VII-2015, Qi-kun Bai & Lu Qiu leg.; 4 females, Guangxi Prov., Guiping City, Longtan Park, 31-V–2-VI-2014, Xin-ran Li & Shun-hua Gui leg.; 2 females, Guangxi Prov., Jinxiu County vicinity, Laoshan, 9-VII-2015, Lu Qiu & Qi-kun Bai leg.; 1 male, Yunnan Prov., Longchuan County, 7–8-VI-1981, Zhi-gang Zheng & Ying-shu Xie leg.

##### Distribution.

China (Guangxi, Yunnan).

#### Rhabdoblatta rattanakiriensis

Taxon classificationAnimaliaBlattodeaBlaberidae

Anisyutkin, 1999

Figures 8I–L , 12G

Rhabdoblatta rattanakiriensis Anisyutkin, 1999: 253.

##### Measurements (mm).

Female, overall length: 28.0–29.0.

##### Female.

Female different from male. Body slightly larger. Color of female darker. Middle of every abdominal sterna with brown stripes (Figure 8I–L).

##### Female genitalia.

Moderately sclerotized. Ovipositor back to brood sac. Tergal process of the eighth abdominal tergite slender, from the base to the end gradually narrowing, length ca. 1/2 of tergal process of the ninth abdominal tergite. Tergal process of the ninth abdominal tergite weakly sclerotized, linked with tergal process of the eighth abdominal tergite. First valves of ovipositor slender, apex membranous, inner margin with fine bristles. Second valves of ovipositor tube-shaped, completely covered by the first valves of ovipositor. Third valves of ovipositor slightly wider, length shorter than the first valves of ovipositor. Gonangulum weakly sclerotized. Sclerotized lobes of the second and third pairs of valves nearly rectangular. Anterior arch of second valvifer slender. Basivalvula robust and with semicircular arms, the mid sclerite separate. Vestibular sclerite wide, apical sclerite vestigial. Transverse sclerotized plate absent. Brood sac membranous and without sclerotized section (Figure 12G).

##### Material examined.

26 males and 4 females, Hainan Prov., Wuzhishan Nature Reserve, 18–21-V-2014, Xin-ran Li, Shun-hua Gui & Jian-yue Qiu leg.; 1 male, Hainan Prov., Jianfengling, Mingfenggu, 26-IV-2015, Lu Qiu & Qi-kun Bai leg.; 4 males and 2 females, Hainan Prov., Diaoluoshan Mountain, 916 m, 18-IV-2015, Lu Qiu & Qi-kun Bai leg.

##### Distribution.

China (Hainan).

#### Rhabdoblatta elegans

Taxon classificationAnimaliaBlattodeaBlaberidae

Anisyutkin, 2000

Figures 8M–P , 12H

Rhabdoblatta elegans Anisyutkin, 2000: 190.

##### Measurements (mm).

Female, overall length: 38.3–42.7.

##### Female.

Female similar to male but slightly larger (Figure 8M–P).

##### Female genitalia.

Moderately sclerotized. Ovipositor back to brood sac. Tergal process of the eighth abdominal tergite slender, from the base to the end gradually narrowing, length ca. 1/2 of tergal process of the ninth abdominal tergite. Tergal process of the ninth abdominal tergite robust, linked with tergal process of the eighth abdominal tergite. First valves of ovipositor slender, apex membranous, inner margin with fine bristles. Second valves of ovipositor tube-shaped, completely covered by the first valves of ovipositor. Third valves of ovipositor slightly wider, length shorter than the first valves of ovipositor. Gonangulum boat-shaped. Sclerotized lobes of the second and third pairs of valves nearly rectangular. Anterior arch of second valvifer slender, middle narrow and both sides wide. Basivalvula robust and with semicircular arms, the mid sclerite incompletely separated. Vestibular sclerite wide, apical sclerite vestigial. Transverse sclerotized plate absent. Brood sac membranous and without sclerotized section (Figure 12H).

##### Material examined.

8 males and 10 females, Yunnan Prov., Jinping County, Maandi Township, Butterfly Valley, 14-V-2015, Jian-yue Qiu leg.; 1 female, Yunnan Prov., Baoshan City, Zaolong, 22-VIII-2015, Xin-ran Li & Zhi-wei Qiu leg.; 1 male, Yunnan Prov., Mengla County, 10-V-2015, Jian-yue Qiu leg.; 1 male, Jiangxi Prov., Fuzhou City, Gaoping Town, Qiayuan Village, 1298 m, 5-V-1980, Yan-bao Qiu leg.; 1 male, Guangxi Prov., Jinzhongshan Mountain, 1-VIII-2014, Jian-hua Huang leg.; 1 male and 1 female, Guangdong Prov., Nanling Nature Reserve, 5–7-VI-2010, unknown.

##### Distribution.

China (Yunnan, Guangxi, Guangdong, Jiangxi).

#### Rhabdoblatta nigrovittata

Taxon classificationAnimaliaBlattodeaBlaberidae

Bey-Bienko, 1954

Figures 9A–D , 12I

Rhabdoblatta nigrovittata Bey-Bienko, 1954: 21; Princis 1967: 664; Feng, Guo et Wu 1997: 90; Anisyutkin 2003: 550.

##### Measurements (mm).

Female, overall length: 43.0–45.0.

##### Female.

**Figure 9.**
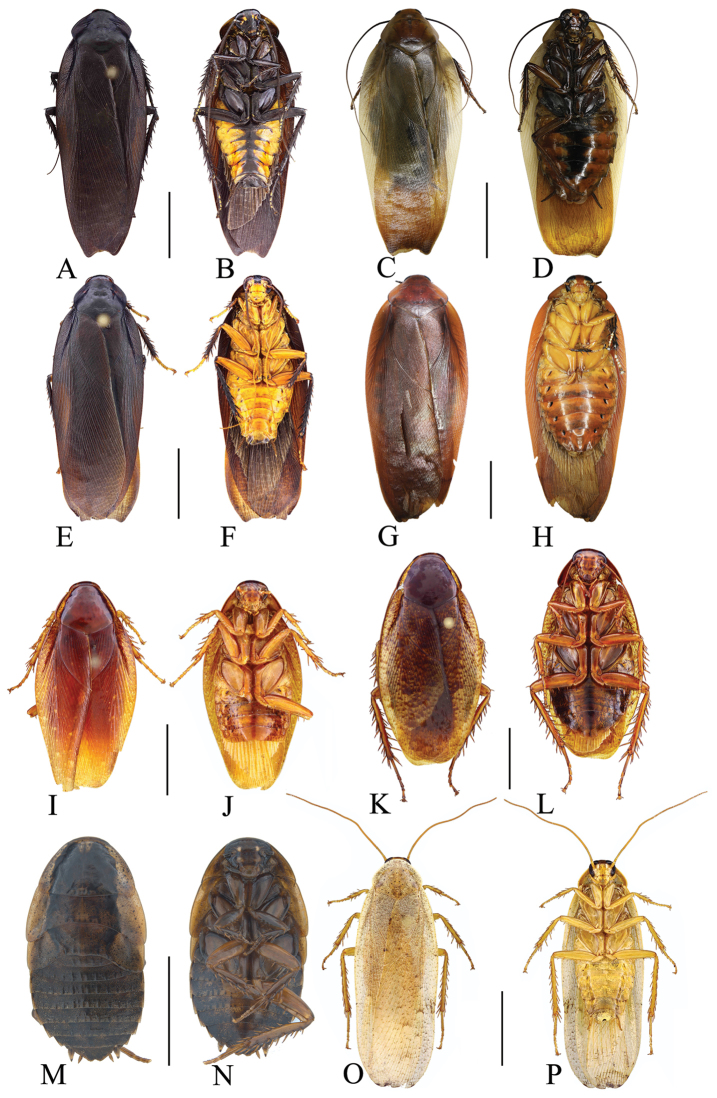
**A–D***Rhabdoblattanigrovittata* Bey-Bienko, 1954: **A, B** male **C, D** female **E–H***Rhabdoblattasimulans* Anisyutkin, 2000: **E, F** male **G, H** female **I–N***Rhabdoblattamarginata* Bey-Bienko, 1969: **I, J** male **K, L** female **M, N** nymph **O, P***Rhabdoblattasinuata* Bey-Bienko, 1958: **O, P** male. Scale bars: 1.0 cm.

Female slightly different from male and larger. Body pale yellow. Vertex and frons yellowish brown. Stripes on the sterna same as male (Figure 9A–D).

##### Female genitalia.

Moderately sclerotized. Ovipositor back to brood sac. Tergal process of the eighth abdominal tergite obviously vestigial, sharp and slender, length ca. 1/3 of tergal process of the ninth abdominal tergite. Tergal process of the ninth abdominal tergite robust, linked with the ninth tergum. First valves of ovipositor with apex membranous, inner margin with fine bristles. Second valves of ovipositor tube-shaped, completely covered by the first valves of ovipositor. Third valves of ovipositor slightly wider, length slightly shorter than the first valves of ovipositor. Gonangulum and sclerotized lobes of the second and third pairs of valves nearly triangular. Anterior arch of second valvifer slender, middle narrow and both sides wide. Basivalvula robust and with semicircular arms, the mid sclerite incompletely separated. Vestibular sclerite wide and robust. Transverse sclerotized plate bowknot-shaped. Brood sac membranous and without sclerotized section (Figure 12I).

##### Material examined.

3 females, Hunan Prov., Mangshan Forest Park, 11–12-VII-2015, Zhi-wei Qiu & Yong-quan Zhao leg.; 3 females and 1 male, Guangxi Prov., Jinxiu County, Shengtangshan Mountain, 12-VII-2015, Lu Qiu & Qi-kun Bai leg.; 1 male, Guangdong Prov., Nanling Nature Reserve, 6–7-VI-2014, Cheng-hui Zhan leg.; 4 females, Chongqing City, Simianshan Mountain, Feilongmiao Temple, 5–6-VI-2015, Lu Qiu & Qi-kun Bai leg.; 10 males and 2 females, Guizhou Prov., Kuankuoshui Nature Reserve, Baishaogou, 4–5-VI-2010, Jia-jia Zhao leg.; 2 males, Zhejiang Prov., Tianmushan Mountain, 7–10-VI-2016, Lian Chen leg.; 1 male and 1 female, Sichuan Prov., Luzhou City, Gulin County, Guihua Township, Lou’e Village, 19-VII-2014, by light trap, Lu Qiu leg.; 1 female, Sichuan Prov., Leshan City, E’meishan Mountain, Sangenqiao, 1124 m, 22-VIII-2017, by light trap, Lu Qiu leg.; 1 female, Guangxi Prov., Jiuxiu City, Dayaoshan Nature Reserve, Hekou Reserve Station, 8-VII-2015, Lu Qiu & Qi-kun Bai leg.; 1 female, Guangxi Prov., Wuming County, Damingshan Mountain, 2-VII-2015, Qi-kun Bai & Lu Qiu leg.; 1 male and 1 female, Hubei Prov., Enshi City, Qizimeishan Mountain, 21-VI-2012, Mao Ye leg.

##### Distribution.

China (Hunan, Hubei, Guangdong, Sichuan, Chongqing, Guizhou, Zhejiang, Guangxi, Yunnan, Fujian).

#### Rhabdoblatta simulans

Taxon classificationAnimaliaBlattodeaBlaberidae

Anisyutkin, 2000

Figures 9E–H , 12J

Rhabdoblatta simulans Anisyutkin, 2000: 191.

##### Measurements (mm).

Female, overall length: 42.7–43.1.

##### Female.

Female slightly different from male and larger. Vertex and area of interocellus yellowish brown (Figure 9E–H).

##### Female genitalia.

Moderately sclerotized. Ovipositor back to brood sac. Tergal process of the eighth abdominal tergite obvious and slender, length ca. 1/2 of tergal process of the ninth abdominal tergite. Tergal process of the ninth abdominal tergite robust, linked with the ninth tergum. First valves of ovipositor with apex wide and cambered, base thin. Second valves of ovipositor tube-shaped, completely covered by the first valves of ovipositor. Third valves of ovipositor slightly wider, length shorter than the first valves of ovipositor. Gonangulum boat-shaped. Sclerotized lobes of the second and third pairs of valves irregular. Anterior arch of second valvifer slender. Basivalvula with semicircular arms, mid sclerite incompletely separated. Vestibular sclerite membranous. Transverse sclerotized plate nearly circular. Brood sac membranous and without sclerotized section (Figure 12J).

##### Material examined.

1 male and 1 female, Yunnan Prov., Yingjiang County, Tongbiguan Township, 1345 m, 31-V-2018, Lu Qiu & Wen-bo Deng leg.; 1 female, Yunnan Prov., Yingjiang County, around Tongbiguan Township, 1345 m, 2-VI-2018, Lu Qiu & Wen-bo Deng leg.; 2 males and 1 female, Xizang Prov., Medog County, 3-VIII-2017, unknown.

##### Distribution.

China (Yunnan, Xizang).

#### Rhabdoblatta marginata

Taxon classificationAnimaliaBlattodeaBlaberidae

Bey-Bienko, 1969

Figures 9I–N , 12K

Rhabdoblatta marginata Bey-Bienko, 1969: 843; Bey-Bienko 1970: 363; Princis 1971: 1158; Anisyutkin 2003: 547.

##### Measurements (mm).

Female, overall length: 32.5–34.0.

##### Female.

Female larger than male and color slightly darker (Figure 9I–L).

##### Female genitalia.

Moderately sclerotized. Ovipositor back to brood sac. Tergal process of the eighth abdominal tergite obviously vestigial, length ca. 1/2 of tergal process of the ninth abdominal tergite. Tergal process of the ninth abdominal tergite robust, linked with the ninth tergum. First valves of ovipositor with apex membranous, inner margin with fine bristles. Second valves of ovipositor tube-shaped, completely covered by the first valves of ovipositor. Third valves of ovipositor slightly wider, length shorter than the first valves of ovipositor. Gonangulum boat-shaped. Sclerotized lobes of the second and third pairs of valves nearly triangle. Anterior arch of second valvifer slender. Basivalvula with semicircular arms, the mid sclerite incompletely separated. Vestibular sclerite slender, the mid sclerite membranous. Transverse sclerotized plate nearly circular. Brood sac membranous and without sclerotized section (Figure 12K).

##### Nymph.

Body dark brown. Pronotum black, lateral area with a yellow stripe and black spots on the surface. Abdominal terga dark brown, with short and bar-shaped convexity. Abdominal sterna dark brown and with scattered black spots on the surface (Figure 9M, N).

##### Material examined.

8 males and 8 females, Hainan Prov., Wuzhishan Nature Reserve, 18–21-V-2014, Shun-hua Gui, Xin-ran Li & Jian-yue Qiu leg.; 1 female and 1 nymph, Hainan Prov., Wuzhishan Mountain, 920 m, 21-XI-2013, Yan Shi leg.; 3 nymphs, Hainan Prov., Baoting County, Maogan Township, 549–776 m, 11–12-IV-2015, Lu Qiu & Qi-kun Bai leg.; 1 male and 1 female, Hainan Prov., Limushan Mountain, 15-IV-2015, Zhi-wei Qiu & Xin-ran Li leg.; 1 male, Hainan Prov., Changjiang County, Bawangling, Yajia, 29-VI-2015, Lu Qiu & Qi-kun Bai leg.; 10 males and 10 females, Guangdong Prov., Conghua District, Liuxihe National Forest Park, Wuzhishan Scenic, 7–8-VII-2015, Zhi-wei Qiu & Yong-quan Zhao leg.; 4 males and 5 females, Guangxi Prov., Shangsi County, Shiwandashan Forest Park, 28-VI-2015, Lu Qiu & Qi-kun Bai leg.; 1 male, Guangxi Prov., Jinxiu County, Shengtangshan Mountain, 4–5-VI-2014, Shun-hua Gui & Xin-ran Li leg.

##### Distribution.

China (Hainan, Guangdong, Guangxi).

#### Rhabdoblatta sinuata

Taxon classificationAnimaliaBlattodeaBlaberidae

Bey-Bienko, 1958

**Figure 10.**
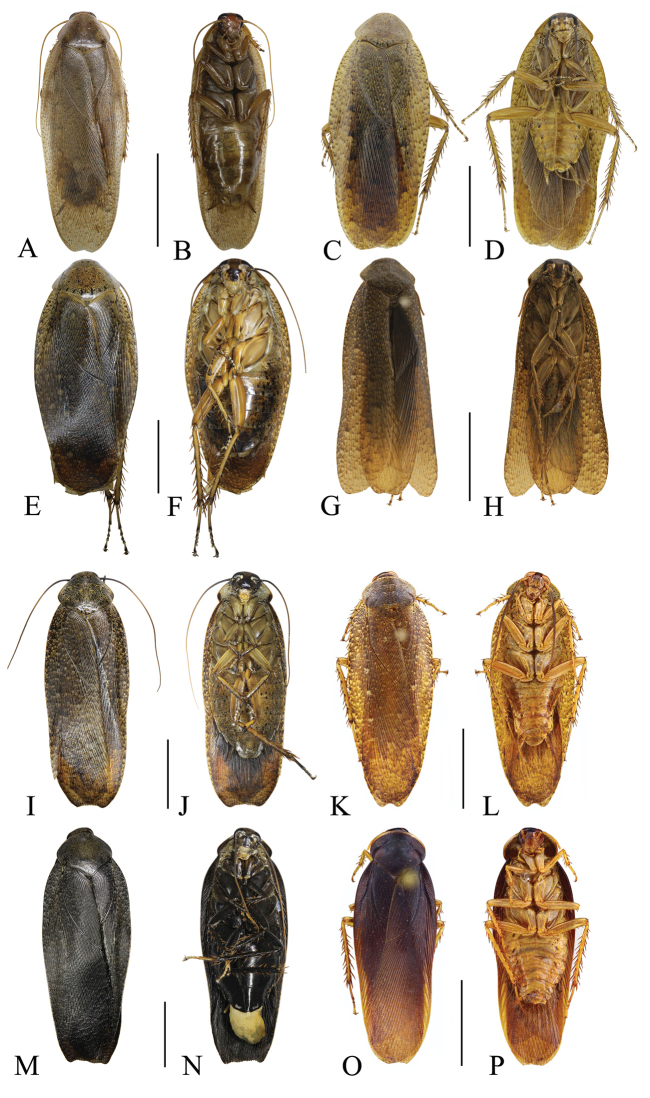
**A, B***Rhabdoblattasinuata* Bey-Bienko, 1958: **A, B** female **C–F***Rhabdoblattamascifera* Bey-Bienko, 1969: **C, D** male **E, F** female **G–J***Rhabdoblattaincisa* Bey-Bienko, 1969: **G, H** male **I, J** female **K–N***Rhabdoblattakrasnovi* (Bey-Bienko, 1969): **K, L** male **M, N** female **O, P***Rhabdoblattamelancholica* (Bey-Bienko, 1954): **O, P** male. Scale bars: 1.0 cm.

Figures 9O, P , 10A, B , 12L

Rhabdoblatta sinuata Bey-Bienko, 1958: 593; Bey-Bienko 1970: 368; Princis 1967: 675; Anisyutkin 2003: 552.

##### Measurements (mm).

Female, overall length: 35.0–36.0.

##### Female.

Female similar to male but slightly larger (Figure 9O, P, Figure 10A, B).

##### Female genitalia.

Moderately sclerotized. Ovipositor extending toward brood sac. Tergal process of the eighth abdominal tergite obviously vestigial, from the base to the end gradually more narrow, length ca. 1/3 of tergal process of the ninth abdominal tergite. Tergal process of the ninth abdominal tergite wide, linked with the ninth tergum. First valves of ovipositor with apex membranous, inner margin with fine bristles. Second valves of ovipositor tube-shaped, completely covered by the first valves of ovipositor. Third valves of ovipositor slightly wider, length shorter than the first valves of ovipositor. Gonangulum boat-shaped. Sclerotized lobes of the second and third pairs of valves crescent-shaped. Anterior arch of second valvifer slender, middle narrow and both sides wide. Basivalvula with semicircular arms, the mid sclerite incompletely separated. Vestibular sclerite weakly scleritized, the mid sclerite nearly membranous. Transverse sclerotized plate nearly semicircle. Brood sac membranous and without sclerotized section (Figure 12L).

##### Material examined.

12 males and 2 females, Yunnan Prov., Jinping County, Maandi Village, Butterfly Valley, 15-V-2015, Jian-yue Qiu leg.; 5 males and 1 female, Sichuan Prov., Panzhihua City, Daheishan Forest Park, 20–21-V-2011, unknown; 5 males, Guangdong Prov., Nanling Nature Reserve, 5–7-VI-2010, Ke-liang Wu & Jia-jia Wu leg.

##### Distribution.

China (Yunnan, Sichuan, Guangdong).

#### Rhabdoblatta mascifera

Taxon classificationAnimaliaBlattodeaBlaberidae

Bey-Bienko, 1969

Figures 10C–F , 12M

Rhabdoblatta mascifera Bey-Bienko, 1969: 844; Bey-Bienko 1970: 368; Princis 1971: 1158; Anisyutkin 2003: 547.

##### Measurements (mm).

Female, overall length: 32.4.

##### Female.

Female slightly bigger and frons dark brown (Figure 10C–F).

##### Female genitalia.

Moderately sclerotized. Ovipositor back to brood sac. Tergal process of the eighth abdominal tergite from the base to the end gradually more narrow, length ca. 1/2 of tergal process of the ninth abdominal tergite. Tergal process of the ninth abdominal tergite linked to the ninth tergum. First valves of ovipositor with apex membranous, inner margin with fine bristles. Second valves of ovipositor tube-shaped, completely covered by the first valves of ovipositor. Third valves of ovipositor slightly wider, length shorter than the first valves of ovipositor. Gonangulum boat-shaped. Sclerotized lobes of the second and third pairs of valves irregular. Anterior arch of second valvifer slender. Basivalvula with semicircular arms, the mid-sclerite incompletely separated. Vestibular sclerite nearly membranous. Transverse sclerotized plate absent. Brood sac membranous and without sclerotized section (Figure 12M).

##### Material examined.

2 males, Yunnan Prov., Xishuangbanna, Mengla County, Shangyong Town, Longmen Village, 8–9-V-2015, Jian-yue Qiu leg.; 1 female, Yunnan Prov., Xishuangbanna, Menglun Town, Xishuangbanna Tropical Botanical Garden, Gouguyulin, 27-V-2016, Zhi-wei Qiu & Lu Qiu leg.

##### Distribution.

China (Yunnan).

#### Rhabdoblatta incisa

Taxon classificationAnimaliaBlattodeaBlaberidae

Bey-Bienko, 1969

Figures 10G–J , 12N

Rhabdoblatta incisa Bey-Bienko, 1969: 843; Princis 1971: 1158; Feng, Guo et Wu 1997: 95; Anisyutkin 2003: 547.

##### Measurements (mm).

Female, overall length: 33.2.

##### Female.

Female similar to male but slightly larger (Figure 10G–J).

##### Female genitalia.

Moderately sclerotized. Ovipositor back to brood sac. Tergal process of the eighth abdominal tergite obvious, gradually narrowing from the base to the end, length ca. 1/2 of tergal process of the ninth abdominal tergite. Tergal process of the ninth abdominal tergite robust, linked with the ninth tergum. First valves of ovipositor with apex membranous, inner margin with fine bristles. Second valves of ovipositor tube-shaped, completely covered by the first valves of ovipositor. Third valves of ovipositor slightly wider, whose length slightly shorter than the first valves of ovipositor. Gonangulum irregular. Sclerotized lobes of the second and third pairs of valves boat-shaped. Anterior arch of second valvifer slender, middle narrow and both sides wide. Basivalvula with semicircular arms, the mid sclerite incompletely separated. Vestibular sclerite weakly sclerotized, apical sclerite vestigial. Transverse sclerotized plate membranous. Brood sac membranous and without sclerotized section (Figure 12N).

##### Material examined.

20 males and 2 females, Yunnan Prov., Xinping County, Ailaoshan Mountain, Yaonan Village, 12-V-2016, Lu Qiu & Zhi-wei Qiu leg.; 1 female, Yunnan Prov., Pingbian County, Daweishan Mountain, Qianjin Village, 17-V-2016, Lu Qiu & Zhi-wei Qiu leg.

##### Distribution.

China (Yunnan, Guizhou, Guangxi).

#### Rhabdoblatta krasnovi

Taxon classificationAnimaliaBlattodeaBlaberidae

(Bey-Bienko, 1969)

Figures 10K–N , 12O

Stictolomapra krasnovi Bey-Bienko, 1969: 536; Princis 1971: 1159.
Rhabdoblatta krasnovi Anisyutkin, 2003: 552.

##### Measurements (mm).

Female, overall length: 35.5.

##### Female.

Female different from male and slightly larger. Body dark brown, darker than male. Frons dark brown. Tarsus and pretarsus yellowish, the remaining is dark brown (Figure 10K–N).

##### Female genitalia.

Moderately sclerotized. Ovipositor back to brood sac. Tergal process of the eighth abdominal tergite slender, gradually narrowing from the base to the end, length ca. 1/2 of tergal process of the ninth abdominal tergite. Tergal process of the ninth abdominal tergite robust, linked with the ninth tergum. First valves of ovipositor with apex membranous, inner margin with fine bristles. Second valves of ovipositor tube-shaped, completely covered by the first valves of ovipositor. Third valves of ovipositor wider, length slightly shorter than the first valves of ovipositor. Gonangulum boat-shaped. Sclerotized lobes of the second and third pairs of valves flake-shaped. Anterior arch of second valvifer slender, with concavity in the middle. Basivalvula with semicircular arms, the mid sclerite incompletely separated. Vestibular sclerite membranous, apical sclerite vestigial. Transverse sclerotized plate absent. Brood sac membranous and without sclerotized section (Figure 12O).

##### Material examined.

7 males and 1 female, Yunnan Prov., Pingbian County, Daweishan Mountain, Hongqi Reservoir, 15-V-2016, Lu Qiu & Zhi-wei Qiu leg.; 10 males and 2 females, Yunnan Prov., Jinping County, Maandi Village, Butterfly Valley, 14-V-2015, Jian-yue Qiu leg.; 1 male, Guangxi Prov., Guilin City, Huaping Nature Reserve, Hongtan, 11-VI-1963, Ji-kun Yang leg.; 1 male, Chongqing City, Jiangjing, Simianshan Mountain, 1000 m, 20-V-2007, Wei-wei Zhang leg.

##### Distribution.

China (Yunnan, Guangxi, Chongqing).

#### Rhabdoblatta melancholica

Taxon classificationAnimaliaBlattodeaBlaberidae

(Bey-Bienko, 1954)

**Figure 11.**
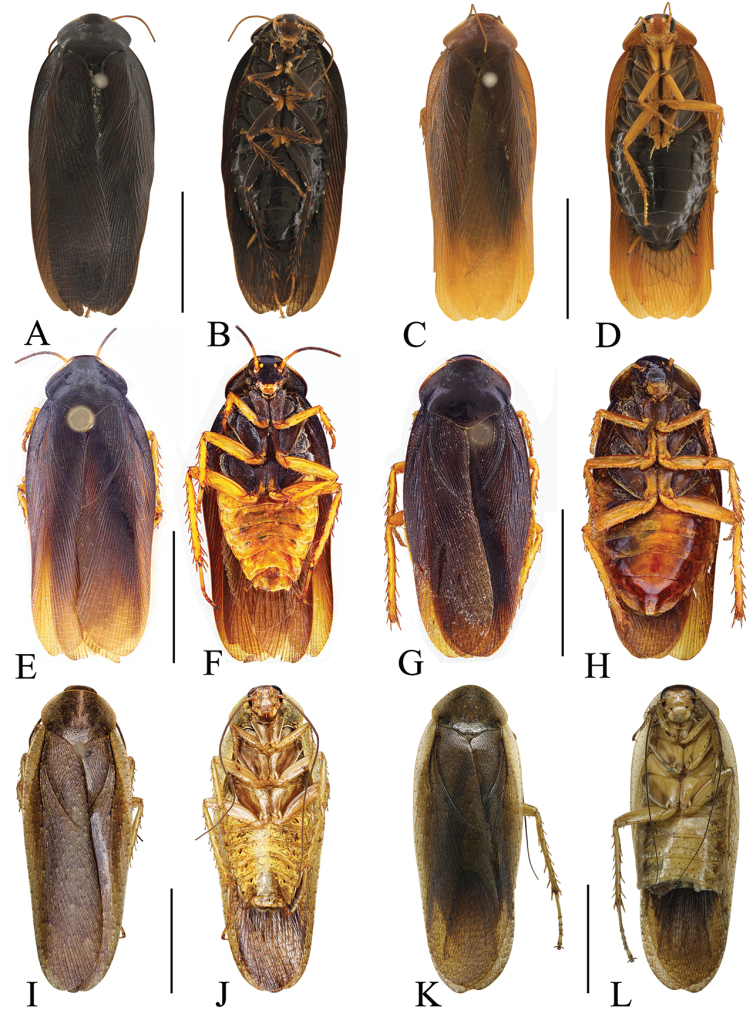
**A–D***Rhabdoblattamelancholica* (Bey-Bienko, 1954): **A–D** female **E–H***Rhabdoblattabicolor* (Guo, Liu et Li, 2011): **E, F** male, **G, H** female **I–L***Rhabdoblattasaussurei* (Kirby, 1903): **I, J** male **K, L** female. Scale bars: 1.0 cm.

**Figure 12.**
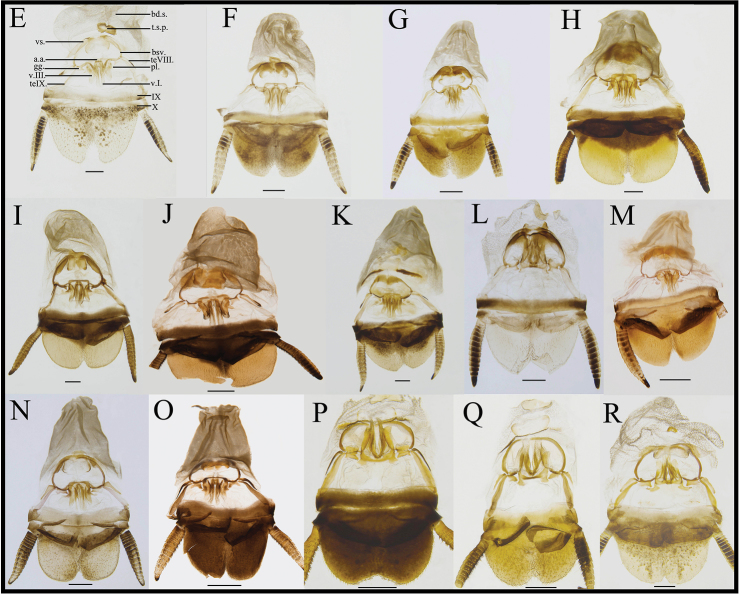
**E–R** Female genitalia. **E***Rhabdoblattamonticola* (Kirby, 1903) **F***Rhabdoblattaatra* Bey-Bienko, 1970 **G***Rhabdoblattarattanakiriensis* Anisyutkin, 1999 **H***Rhabdoblatta*elegans Anisyutkin, 2000 **I***Rhabdoblattanigrovittata* Bey-Bienko, 1954 **J***Rhabdoblattasimulans* Anisyutkin, 2000 **K***Rhabdoblattamarginata* Bey-Bienko, 1969 **L***Rhabdoblattasinuata* Bey-Bienko, 1958 **M***Rhabdoblattamascifera* Bey-Bienko, 1969 **N***Rhabdoblattaincisa* Bey-Bienko, 1969 **O***Rhabdoblattakrasnovi* (Bey-Bienko, 1969) **P***Rhabdoblattamelancholica* (Bey-Bienko, 1954) **Q***Rhabdoblattabicolor* (Guo, Liu et Li, 2011) **R***Rhabdoblattasaussurei* (Kirby, 1903). Scale bars: 1.0 mm.

Figures 10O, P , 11A–D , 12P

Stictolomapra melancholica Bey-Bienko, 1954: 21; Bey-Bienko 1957: 901; Princis 1967: 686.
Rhabdoblatta melancholica , Anisyutkin, 2003: 550.

##### Measurements (mm).

Female, overall length: 20.3–22.0.

##### Female.

Female similar to male. But individual color variable (Figures 10O, P, 11A–D).

##### Female genitalia.

Moderately sclerotized. Ovipositor extending toward to brood sac. Tergal process of the eighth abdominal tergite slender, length ca. 1/2 of tergal process of the ninth abdominal tergite. Tergal process of the ninth abdominal tergite robust, linked with the ninth tergum. First valves of ovipositor with apex membranous, inner margin with fine bristles. Second valves of ovipositor tube-shaped, completely covered by the first valves of ovipositor. Third valves of ovipositor wider, length slightly shorter than the first valves of ovipositor. Gonangulum irregular. Sclerotized lobes of the second and third pairs of valves flake-shaped. Anterior arch of second valvifer narrow in middle and both sides wider. Basivalvula with semicircular arms, the mid-sclerite separate. Vestibular sclerite membranous, apical sclerite vestigial. Transverse sclerotized plate semicircular. Brood sac membranous and without sclerotized section (Figure 12P).

##### Material examined.

2 males, Guangxi Prov., Jinxiu County, Dayaoshan Nature Reserve, Hekou Reserve Station, 6–7-VII-2015, Lu Qiu & Qi-kun Bai leg.; 1 female, Guangxi Prov., Jinxiu County, Yinshan Park, 16–17-VII-2015, Lu Qiu & Qi-kun Bai leg.; 1 male, Guizhou Prov., Kuankuoshui Nature Reverse, Baishaogou, 5-VI-2010, Ke-liang Wu & Jia-jia Zhao leg.; 2 males and 1 female, Chongqing Prov., Simianshan Mountain, Ertai, 20-VI-2014, Hao Xu leg.; 10 males and 5 females, Sichuan Prov., Dujiangyan City, Qingchengshan Town, 19-V-2014, Lu Qiu; 50 males, Hubei Prov., Dabieshan Mountain, Taohuachong, 604 m, 27-VI-2014, Xin-ran Li & Yan Shi leg.; 10 females, Zhejiang Prov., Lin’an City, Tianmu Village, 23-VII-2016, Lu Qiu & Zhi-wei Qiu leg.; 2 females, Anhui Prov., Huangshan City, Tangkou Town, 10-VII-2014, Xin-ran Li & Jian-yue Qiu leg.; 8 males and 1 female, Hainan Prov., Diaoluoshan Mountain, 18-IV-2015, Lu Qiu & Qi-kun Bai leg.; 16 males and 2 females, Hainan Prov., Diaoluoshan Mountain, 916 m, 16-IV-2015, Lu Qiu & Qi-kun Bai leg.; 2 males, Fujian Prov., Wuyishan Mountain, 10-VI-1980, Shi-yang Xia leg.; 1 male, Guangdong Prov., Nankunshan Mountain, 15-VI-1981, Qin-jin Liu & Xue-feng Li leg.; 1 male, Jiangxi Prov., Jinggangshan Mountain, 23-V-1981, Jin Liu & Yao Liu leg.; 1 male, Shaanxi Prov., Foping County, 890 m, 26-VI-1999, You-wei Zhang leg.; 1 male, Gansu Prov., Kangxian County, Yangba Town, 1020 m, 10-VII-1999, Hong-jian Wang leg.; l male, Huan Prov., Hengshan Mountain, Mojingtai, 21-VI-1963, Ji-kun Yang leg.; 1 male, Yunnan Prov., Cangyuan County, 750 m, 19-V-1980, Hong-xing Li leg.

##### Distribution.

China (Fujian, Guangxi, Guangdong, Guizhou, Chongqing, Sichuan, Jiangxi, Shaanxi, Gansu, Hunan, Hubei, Zhejiang, Anhui, Yunnan, Hainan).

**Figure 13.**
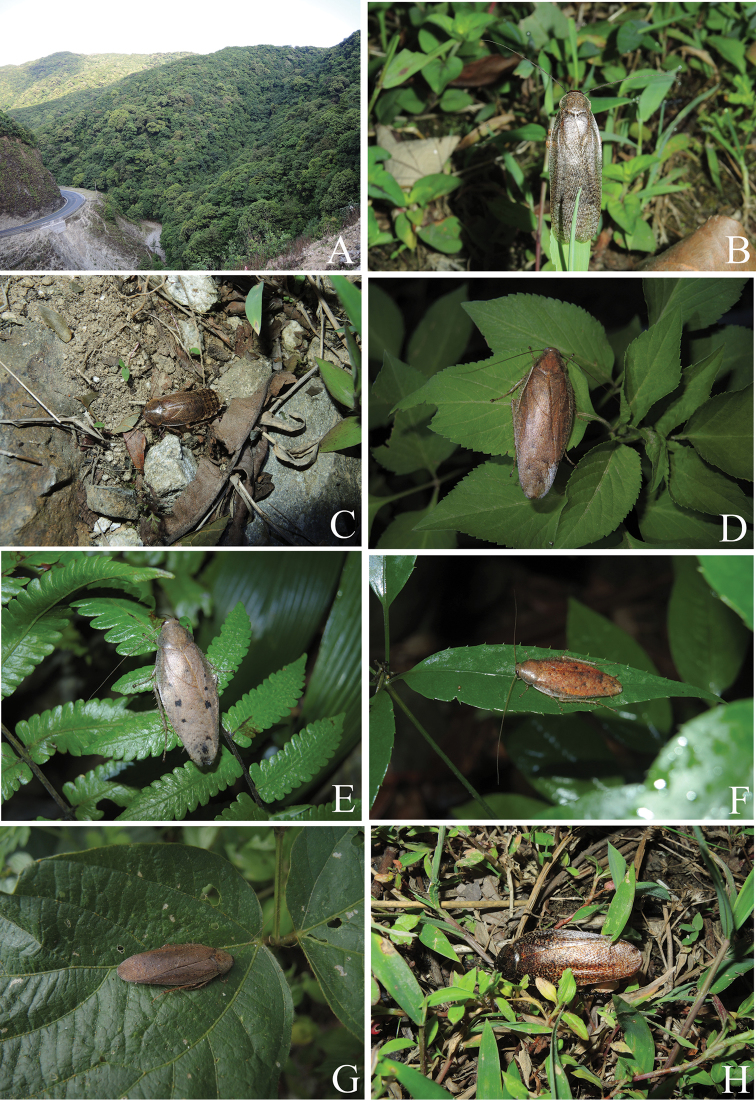
**A** Habitat of Ailao Mountain, Yunnan Prov. **B** male of *Rh.similsinuata* sp. n. from Ailao Mountain **C** female of *Rh.similsinuata* sp. n. from Ailao Mountain **D***Rh.monticola* (Kirby, 1903) from Shiwandashan National Forest Park, Guangxi Prov. **E, F** male of *Rh.ecarinata* sp. n. from Yinggeling Nature Reserve, Hainan Prov. **G***Rh.atra* Bey-Bienko, 1970 from Dayaoshan Nature Reserve, Guangxi Prov. **H***Rh.densimaculata* sp. n. from Ailao Mountain, Yunnan Prov. All the photographs were taken by Lu Qiu.

#### Rhabdoblatta bicolor

Taxon classificationAnimaliaBlattodeaBlaberidae

(Guo, Liu & Li, 2011)

Figures 11E–H , 12Q

Rhabdoblatta bicolor Guo, Liu et Li, 2011: 723.

##### Measurements (mm).

Female, overall length: 19.0–22.5.

##### Female.

Female similar to male (Figure 11E–H).

##### Female genitalia.

Moderately sclerotized. Ovipositor extends toward brood sac. Tergal process of the eighth abdominal tergite slender, length ca. 1/3 of tergal process of the ninth abdominal tergite. Tergal process of the ninth abdominal tergite robust, linked with the ninth tergum. First valves of ovipositor with apex membranous, inner margin with fine bristles. Second valves of ovipositor tube-shaped, completely covered by the first valves of ovipositor. Third valves of ovipositor wide and flat, length slightly shorter than the first valves of ovipositor. Gonangulum irregular. Sclerotized lobes of the second and third pairs of valves flake-shaped. Anterior arch of second valvifer narrow in middle and both sides wide. Basivalvula with semicircular arms, the mid-sclerite separate. Vestibular sclerite membranous, apical sclerite vestigial. Transverse sclerotized plate semicircle. Brood sac membranous and without sclerotized section (Figure 12Q).

##### Material examined.

3 males, Zhejiang Prov., Jiangshan City, Shuangxikou Village, 26–27-V-2017, Xin-ran Li, Li-li Wang & Meng Li leg.; 3 males and 1 female, Zhejiang Prov., Jiangshan City, Shuangxikou Village, 26–27-V-2017, Hua Zhang leg.; 1 female, Anhui Prov., Huangshan City, Tangkou Town, 10-VII-2014, Xin-ran Li & Jian-yue Qiu leg.; 1 female, Chongqing Ciry, Pengshui County, Taiyuan Village, 850 m, 10-VII-1989, Long-long Yang leg.; 1 female, Guizhou Prov., Qiandongnan Zhou, Shibing County, Shamuhe, 19-VI-1981, unknown; 1 female, Guangxi Prov., Jinxiu County, Wangshanzhuang, 20-V-1999, Fu-sheng Huang leg.

##### Distribution.

China (Chongqing, Guizhou, Zhejiang, Anhui, Guangxi).

#### Rhabdoblatta saussurei

Taxon classificationAnimaliaBlattodeaBlaberidae

(Kirby, 1903)

Figure 11I–L , 12R

Stictolomapra saussurei , Princis, 1952: 38; Bey-Bienko 1957: 901; Princis 1967: 683.
Rhabdoblatta saussurei , Anisyutkin, 2003: 555.

##### Measurements (mm).

Female, overall length: 38.0–43.0.

##### Female.

Female similar to male but slightly larger (Figure 11I–L).

##### Female genitalia.

Moderately sclerotized. Ovipositor toward to brood sac. Tergal process of the eighth abdominal tergite vestigial, whose length ca. 1/2 of tergal process of the ninth abdominal tergite. Tergal process of the ninth abdominal tergite robust, linked with the ninth tergum. First valves of ovipositor with apex membranous, inner margin with fine bristles. Second valves of ovipositor tube-shaped, completely covered by the first valves of ovipositor. Third valves of ovipositor wide and flat, length slightly shorter than the first valves of ovipositor. Gonangulum boat-shaped. Sclerotized lobes of the second and third pairs of valves nearly crescent-shaped, margin with scattered yellow spots. Anterior arch of second valvifer narrow in middle and both sides wider. Basivalvula with semicircular arms, the mid-sclerite weakly sclerotized and separated. Vestibular sclerite membranous, apical sclerite vestigial. Transverse sclerotized plate small, arc-shaped. Brood sac membranous and without sclerotized section (Figure 12R).

##### Material examined.

1 female, Guangxi Prov., Hechi City, Huanjiang County, Chuanshan Town, Shecun Village, 18–23-VII-2015, Jian-yue Qiu leg.; 1 male, Yunnan Prov., Mengla County, Shangyong Town, Longmen Village, 8–9-V-2015, Jian-yue Qiu leg.; 1 male, Guangdong Prov., Qingyuan City, Lianshan County, 1970, Ping Lin leg.

##### Distribution.

China (Guangxi, Guangdong, Yunnan).

## Discussion

We examined the utility of DNA barcode data in *Rhabdoblatta* species identification. Some morphospecies have no morphological differences between different individuals, but their intraspecific genetic distance is much larger than that of other morphospecies. Some species (*Rh.marginata*, *Rh.melancholica*, *Rh.nigrovittata*, *Rh.sinuata*) confirm this issue. The maximum intraspecific genetic distance in *Rh.marginata* is 8.8%, while Hebert et al. (2003) indicated that divergence values between species are ordinarily greater than 3%. For example, the intraspecific and interspecific genetic distance of ectobiid cockroaches ranged from 0.0 to 7.0% and 4.6 to 30.8% (Che et al. 2017). The thrips is 0.0 to 7.91% and 8.65% to 31.15% (Rebijith et al. 2014) and the mosquitoes is 0 to 1.67% and 2.3 to 21.8% (Wang et al. 2012). And *Rh.marginata* was detected as having six MOTUs in ABGD and 7 MOTUs in GMYC. Morphologically, although expressing a larger genetic distance, all male samples of *Rh.marginata* showed no obvious variation in the shape of the male genitalia, only delicate differences in body color and size could be found. Hence we put forth the following view: there is a possibility of the existence of cryptic species for the following reasons: intraspecific genetic distances of same morphospecies of different regions reach to 8.8% in *Rh.marginata*, 7% in *Rh.nigrovittata*, 7.6% in *Rh.sinuata*, and 8.4% in *Rh.melancholica*; and the phenomenon of cryptic specie is not rare in cockroaches, such as the species of *Cryptocercus* are mainly delimitaed by molecular data and chromosome number (Burnside et al. 1999; Che et al. 2016; Bai et al. 2018). The view can be explored more by other methods, such as the numbers of chromosome, etc., in the future.

Our results show that DNA-based species delimitation methods perform well in detecting sexual dimorphism and in matching adults with nymphs. Five species have sexual dimorphism (differs of body color, size, spots, or other features): *Rh.rattanakiriensis*, *Rh.nigrovittata*, *Rh.simulans*, *Rh.krasnovi* and *Rh.similsinuata* sp. n. Their males and females were successfully matched using DNA barcoding, and also the nymph of *Rh.marginata* was successfully matched with the adult according to DNA data.

## Supplementary Material

XML Treatment for Rhabdoblatta similsinuata

XML Treatment for Rhabdoblatta densimaculata

XML Treatment for Rhabdoblatta gyroflexa

XML Treatment for Rhabdoblatta chaulformis

XML Treatment for Rhabdoblatta maculata

XML Treatment for Rhabdoblatta ecarinata

XML Treatment for Rhabdoblatta monticola

XML Treatment for Rhabdoblatta atra

XML Treatment for Rhabdoblatta rattanakiriensis

XML Treatment for Rhabdoblatta elegans

XML Treatment for Rhabdoblatta nigrovittata

XML Treatment for Rhabdoblatta simulans

XML Treatment for Rhabdoblatta marginata

XML Treatment for Rhabdoblatta sinuata

XML Treatment for Rhabdoblatta mascifera

XML Treatment for Rhabdoblatta incisa

XML Treatment for Rhabdoblatta krasnovi

XML Treatment for Rhabdoblatta melancholica

XML Treatment for Rhabdoblatta bicolor

XML Treatment for Rhabdoblatta saussurei
